# Stable and Lead‐Free Perovskite Hemispherical Photodetector for Vivid Fourier Imaging

**DOI:** 10.1002/advs.202414430

**Published:** 2024-12-24

**Authors:** Chenglong Li, Weijun Li, Wei Qu, Haijing Hu, Jia Zong, Haotong Wei

**Affiliations:** ^1^ State Key Laboratory of Supramolecular Structure and Materials College of Chemistry Jilin University Changchun 130012 P.R. China; ^2^ Optical Functional Theragnostic Joint Laboratory of Medicine and Chemistry The First Hospital of Jilin University Changchun 130012 P.R. China

**Keywords:** color imaging, filterless, halide perovskites, harsh condition, hemispherical photodetectors

## Abstract

The filterless single‐pixel imaging technology is anticipated to hold tremendous competitiveness in diverse imaging applications. Nevertheless, achieving single‐pixel color imaging without a filter remains a formidable challenge. Here a lead‐free perovskite hemispherical photodetector is reported for filterless single‐pixel color imaging. Through passivating the active vacancy and antisite defects with functional chemical groups, the trap density of the perovskite film can be dramatically reduced to half of the pristine one, which results in a stable device with 0.69 nA standard deviation (SD) of the current fluctuations for over 10 000 s. The exceptional stability and remarkable low light response capability of passivated lead‐free perovskite devices ensure reliable and repeated distinguishment to high‐frequency color information of Fourier patterns. High‐resolution color imaging with 256 × 256 pixels can be achieved by integrating the Bayer template with Fourier patterns and designing a corresponding color restoration algorithm. Notably, its imaging quality surpasses commercial silicon photomultiplier tubes (SiPMs) under low light intensities or high‐temperature conditions as demonstrated by the larger signal‐to‐noise ratio (SNR). This work presents a novel approach for applying perovskite photodetectors in single‐pixel color imaging, propelling the advancement of filterless color imaging technology.

## Graphical abstract

By passivating active vacancies and anti‐site defects with functional chemical groups using guanidinium thiocyanate, the trap density of CsBi_3_I_10_ perovskite films can be significantly reduced to half of its original density, resulting in the production of stable lead‐free perovskite hemispherical photodetectors. These devices enable single‐pixel filterless high‐resolution color imaging and can meet the requirements for color imaging under complex conditions.

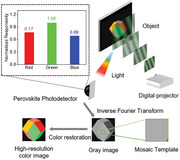

## 1 Introduction

Spurred by the rapid advancement of the Internet of Things and artificial intelligence technology, the information technology industry has entered the era of optical information.^[^ ^1^, ^2^, ^3^ ^]^ The development of optically sophisticated, high‐performance, and cost‐effective photodetectors has become a crucial deployment for the advancement of optical information.^[^ ^4^, ^5^ ^]^ Photodetectors can transform optical signals into electrical signals, streamlining the initial process of information acquisition.^[^ ^6^ ^]^ As a result, they find widespread applications in various domains such as optical imaging,^[^ ^7^, ^8^ ^]^ biological detection,^[^ ^9^, ^10^ ^]^ optical communication,^[^ ^11^, ^12^, ^13^ ^]^ military security,^[^ ^14^ ^]^ etc. However, traditional optical imaging techniques, which are among the most crucial applications, typically require complex filtering mechanisms and numerous pixels, thus increasing the overall complexity and costs.^[^ ^15^ ^]^ For achieving diverse functionalities in different imaging scenarios like wide‐angle^[^ ^16^ ^]^ and night vision,^[^ ^17^ ^]^ smartphones typically incorporate multiple cameras for seamless switching between various contexts or requirements. Regrettably, this strategy featuring complex optical elements and repetitive pixel matrix components leads to a redundant allocation of space and costs. Spatial light modulation‐based single‐pixel imaging technology presents a groundbreaking solution to the conventional multi‐camera dilemma. This innovative technology generates predefined structured light patterns that are subsequently projected onto the subject to be captured.^[^ ^18^, ^19^ ^]^ Fourier imaging is a highly advanced imaging technique that relies on the application of spatial light modulation.^[^ ^20^ ^]^ Through the projection of 2D Fourier orthogonal transformation basis patterns onto the target object, the single‐pixel detector records light intensity values that correspond to the Fourier coefficients of these patterns.^[^ ^21^ ^]^ The collective Fourier coefficients form a comprehensive Fourier spectrum of the target object, facilitating image reconstruction through a meticulous 2D inverse Fourier transform (Figure S1 and S2).^[^ ^22^ ^]^

Nevertheless, traditional Fourier imaging techniques cannot discern colors independently. Consequently, the integration of red, green, and blue lenses becomes imperative to individually capture and extract RGB channels, which are then amalgamated to generate a colorful image. (Figure S3). Although this technique enables the acquisition of colored images, it significantly prolongs the imaging process duration. Moreover, it diminishes the efficacy of capturing spatial and spectral data, potentially leading to color discrepancies that could markedly impair the image quality. This challenge poses a significant barrier to the progress of single‐pixel imaging technology.^[^ ^23^ ^]^ The color information of the Fourier imaging technique lies in the high‐frequency Fourier patterns, and the dense stripes with much similar intensity impose difficulty for single‐pixel photodetector to distinguish. In addition, the predominant methodology in optical imaging revolves around diffuse reflection imagery, wherein light scatters uniformly in all directions.^[^ ^24^, ^25^ ^]^ In the realm of Fourier imaging, the magnitude of luminous flux captured by the photodetectors under identical conditions holds paramount importance for imaging excellence, particularly in dimly lit scenarios. Nevertheless, the majority of ongoing research leans toward employing planar photodetectors.^[^ ^16^ ^]^ In contrast to their planar counterparts, hemispherical photodetectors present a natural advantage for wide‐angle detection within lens‐free systems.^[^ ^26^, ^27^ ^]^ Furthermore, hemispherical photodetectors operate akin to solid immersion lenses, elevating optical power density and enhancing the efficacy of light signal gathering. Although SiPMs are currently the dominant option for advanced photodetectors, the rigid nature of silicon poses a challenge to the intricate manufacturing process of hemispherical photodetectors. Hence, the selection of material for Fourier imaging emerges as a pivotal determinant in dictating imaging proficiency.^[^ ^25^ ^]^

In this article, we report the guanidinium thiocyanate (GTC) passivated cesium bismuth iodide (CsBi_3_I_10_‐GTC), a lead‐free perovskite for hemispherical photodetector through spray‐coating to achieve filterless single‐pixel color imaging. We rationally design a matching color restoration algorithm by combining the Bayer template with the Fourier single‐pixel imaging patterns. The hemispherical photodetector increases the adaptability to spatial scattering light during imaging. The stable device response enables better imaging quality than commercial SiPMs under complex environmental conditions such as low‐light and high‐temperature conditions.

## 2 Results

### 2.1 Reliable Lead‐Free CsBi_3_I_10_ Perovskite Photodetectors

Solution‐processed halide perovskites are good candidates for photon detection due to their excellent and tunable optoelectronic properties and compositions. The selection of appropriate perovskite materials plays a pivotal role in determining the feasibility and quality of imaging. The lead‐free perovskite stands out due to its non‐toxic nature and steadily improved optoelectronic properties. Due to the minute fluctuations in the current signal during the imaging process, Fourier imaging necessitates capturing these subtle variations across a broad dynamic range. Consequently, advanced photodetectors must exhibit both exceptional sensitivity and long‐term stability. To meet the stringent requirements of high‐resolution imaging, we discover that the GTC can serve as a stabilizer for CsBi_3_I_10_ perovskite to stabilize the photodetector performance.

**Figure 1:**
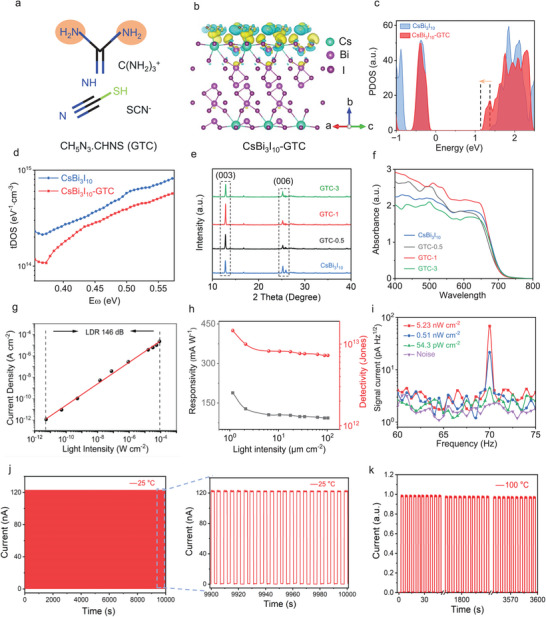
Performance characterization of CsBi_3_I_10_‐GTC photodetectors. a), The chemical structural formula of guanidine thiocyanate (GTC). b), Schematic diagram of differential charge calculation for CsBi_3_I_10_‐GTC. c), The total density of state of the CsBi_3_I_10_‐GTC and CsBi_3_I_10_. d), Trap density of state of perovskite films with and without GTC. e), The evolution of XRD characteristic peaks of CsBi_3_I_10_ and CsBi_3_I_10_‐GTC films. f), UV–vis absorption spectra of GTC and CsBi_3_I_10_‐GTC films with different percentages. g), The I‐P curve of the CsBi_3_I_10_‐GTC photodetector, and the corresponding LDR. h), The light intensity‐dependent responsivity (R) and detectivity (D^*^) of CsBi_3_I_10_ and CsBi_3_I_10_‐GTC photodetectors. i), Signal current under 70 Hz 532 nm light with different irradiances. j), The I‐t curve of the CsBi_3_I_10_‐GTC device under 10 000 s optical switching cycle. k), Time‐dependent photoresponse curves of CsBi_3_I_10_‐GTC photodetectors at 100 °C for 3600 s.

The crystal structure of CsBi_3_I_10_ is shown in Figure S4a. The octahedra formed by metal halides (Bi‐I) are interconnected through face‐sharing and separated by Cs^+^ ions, constituting the basic framework of the crystal. The chemical structure of GTC is shown in **Figures** **1**a and S4b. The C(NH_2_)_3_^+^ (GA^+^) cations of the GTC additives can effectively passivate cation vacancy defects and Bi‐I antisite defects on the bottom surface of the perovskite film through hydrogen bonds and electrostatic interactions between their amino groups (Figure S4c). In addition, SCN^−^ can coordinate with Bi^3+^ through its lone pair of electrons in its linear structure due to its similar chemical behavior to I^−^ (with an ionic radius of 217 pm, close to that of I^−^ at 220 pm), thereby regulating the nucleation and growth processes of CsBi_3_I_10_ (Figure S5). Density functional theory (DFT) calculations were performed on the differential charge analysis of CsBi_3_I_10_‐GTC in Figure 1b. The results suggest that the presence of additives and interfacial charges indicates the potential formation of chemical bonds between the perovskite and the additives, leading to a more stable bulk phase that improves material stability. The total density of states (tDOS) of the CsBi_3_I_10_ and CsBi_3_I_10_‐GTC systems are displayed in Figure 1c. The downward shift of the conduction band minimum induced by the additives is more favorable for electron transport with passivated electron traps. To assess the passivation effect of GTC on perovskite films, the tDOS was calculated and the results are shown in Figure 1d. The tDOS of the CsBi_3_I_10_‐GTC device is much lower than that of the CsBi_3_I_10_ device, indicating that the addition of GTC has a significant passivating effect on the defects in the perovskite films. Furthermore, Fourier transform infrared spectroscopy analysis is carried out in Figure S6a, and the low wavelength number shift of the SCN^−^ groups further confirms the interaction between CsBi_3_I_10_ and GTC. The XRD results in Figure 1e indicate that CsBi_3_I_10_‐GTC perovskite films exhibit enhanced (003) peak intensity compared to the (006) peak, suggesting improved phase purity and crystallinity, consistent with the enlarged grain size of the perovskite film (Figure S6b,c). Figure 1f records the UV–vis spectra of the CsBi_3_I_10_ film with different GTC amounts. It should be noted that the light absorption intensity also reaches the maximum when 1% GTC is added to the CsBi_3_I_10_ film, which improves the device's light‐harvesting efficiency and contributes to the signal current. Meanwhile, the spectral absorption range of CsBi_3_I_10_‐GTC covers almost the entire visible spectrum, meeting the requirements of color imaging (Figure S7).

Perovskite photodetectors were fabricated using an architecture of glass/indium tin oxide (ITO)/SnO_2_/CsBi_3_I_10_ perovskite (with or without the additive GTC)/PTAA/Cr (Figure S8). For diffuse reflection imaging, linear dynamic range (LDR) is a critical parameter. The GTC was crucial for stabilizing the device performance of CsBi_3_I_10_ perovskite, which can significantly broaden the detection range of the incident light intensity (Figure S9a). The LDR of the CsBi_3_I_10_‐GTC photodetector was 146 dB, substantially larger than the 98 dB of the CsBi_3_I_10_ photodetector, equivalent to a two‐order‐of‐magnitude improvement (Figure 1g and Figure S9b). In addition, the CsBi_3_I_10_‐GTC photodetector also demonstrated excellent low‐light detection capability. Its detectivity (D^*^) and responsivity (R) reached 1.47 × 10^13^ Jones and 188.6 mA W^−1^, respectively, at 650 nm and a light intensity of 5 µW cm^−2^ (Figure 1h and Figure S9c), with a minimum detectable intensity of 54.3 pW cm^−2^ (Figure 1i), meeting the requirements for imaging under low‐light conditions. Figure S9d shows the optimized time‐dependent voltage curve of the CsBi_3_I_10_ photodetector, measuring its response time. Its short rise time (13 µs) and fall time (10 µs) are beneficial for reducing imaging time.

Stability is crucial for color imaging and imaging quality. The CsBi_3_I_10_‐GTC photodetector exhibits no obvious degradation after being placed in the air for 30 days (Figure S10a). It also remained black after being immersed in water for 360 s. In contrast, the pristine CsBi_3_I_10_ film turned yellow, further confirming reduced defects due to the incorporation of GTC (Figure S10b). We then inspected the long‐term switching response stability of the device under ambient conditions. Over an operation duration of 10000 s, there was no decay in photocurrent or dark current, indicating excellent stability of the CsBi_3_I_10_‐GTC device in working conditions (Figure 1j and Figure S10c). We also assessed the thermal stability of the CsBi_3_I_10_‐GTC photodetector by performing XRD tests on the CsBi_3_I_10_‐GTC device at different ambient temperatures (Figure S10d). At 150 °C, the intensity of the characteristic (003) peak of the CsBi_3_I_10_ perovskite remained almost unchanged. Furthermore, Figure 1k and Figure S10e depict the typical I‐t curves of the CsBi_3_I_10_‐GTC photodetector and CsBi_3_I_10_ photodetector at 100 °C, showing minimal variation in photocurrent for the CsBi_3_I_10_‐GTC over 3600 s, indicating good thermal stability of the device. Overall, the CsBi_3_I_10_‐GTC perovskite photodetector exhibited excellent long‐term and light stability.

### 2.2 High‐Frequency Domain Determined Color Fourier Imaging

**Figure 2:**
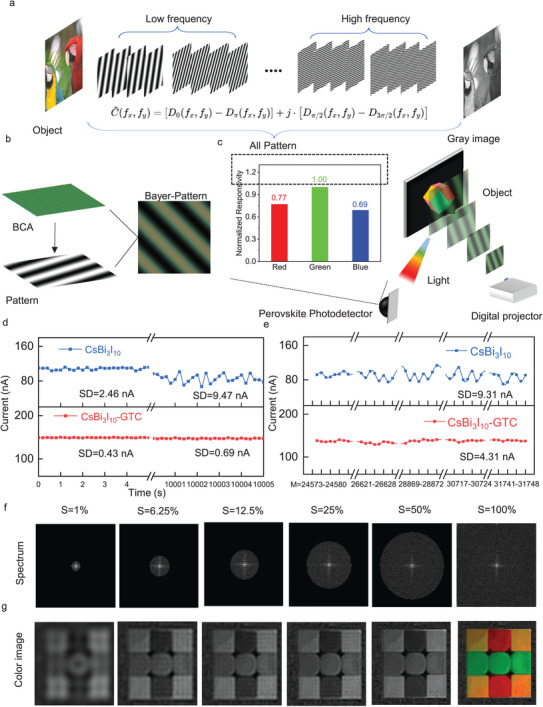
Demonstration of filterless single‐pixel color imaging. a), Fourier single‐pixel imaging principle and spectrum distribution. b), Generation of Fourier color basis patterns and BCFA (Bicolor Fourier Analysis) with different color combinations. c), Experimental schematic diagram of Fourier single‐pixel color imaging based on the Bayer template and the schematic diagram of grayscale image color restoration principle. d), Comparison of current fluctuations between the CsBi_3_I_10_‐GTC photodetector and the CsBi_3_I_10_ photodetector during device operation at different times (first 5 s and last 5 s). e), The current intensity of CsBi_3_I_10_ photodetector and CsBi_3_I_10_‐GTC photodetector under some high‐frequency patterns. f), Fourier coefficient spectrum (log modulus) when the Pattern number (M) increases (from left to right). g), The color images reconstructed corresponding to different numbers of patterns.

In the reported method of Fourier single‐pixel imaging, spatial information can be clearly obtained, which is primarily restricted to the low‐frequency region. The high‐frequency region remains largely untapped, offering an opportunity to encode supplementary information within this range. By exploiting the sparsity of the Fourier space, extra color information can be encoded into the high‐frequency domain of the Fourier spectrum (**Figures** **2**a and S11). To achieve efficient and high‐quality single‐pixel color imaging, we design the prepatterned Bayer template for Fourier single‐pixel color imaging (Figure 2b). The illumination light field in Fourier single‐pixel imaging is modulated using a colored Bayer template, yielding a Fourier spectrum with RGB color channels. The Fourier color basis is generated by performing a point‐by‐point multiplication of the grayscale Fourier spectrum pattern and the Bayer template in the spatial domain. There are various Bayer color array (BCA) patterns. Considering the eye's sensitivity to green, every four pixels are covered with one red, one blue, and two green filters. By combining the CsBi_3_I_10_‐GTC photodetector with the corresponding imaging algorithm, we have achieved Fourier single‐pixel color imaging (Figure 2c). The four‐step phase‐shifting sinusoidal color pattern projected by the projector can be regarded as the light source. A hemispherical photodetector is used to collect the current signals induced by diffuse reflected light under different color patterns. The captured current signals encapsulate spatial and color information pertaining to the 2D image, enabling reconstruction through the application of the inverse Fourier transform algorithm. The color information contained in the reconstructed grayscale 2D image is encoded in a mosaic template. A de‐mosaic algorithm for color restoration is used to reconstruct the color image of the object from the grayscale image (Figure S12).

Since we use a single‐pixel photodetector for imaging, the recording of color is inevitably affected by the material itself. Due to the single‐pixel detector's inconsistent response to light intensity across the RGB spectral channels, the light intensity detection value recorded by the device represents a specific fraction of the combined intensity of the three color lights. Therefore, image color distortion may occur during reconstruction. To address this, we utilize the device's spectral response coefficient for color distortion correction during color restoration, making the reconstructed image's colors more similar to the original object being measured (Figure S13). To select the most suitable BCA for color imaging, we use the aforementioned Fourier color imaging process to numerically simulate the selected image (Figure S14). We chose the GRBG pattern as our BCA for color imaging. By decomposing the GRGB template, we can obtain red, green, and blue encoding templates and perform a Fourier transform (Figure S15). The Fourier transforms of these three templates are linearly independent, guaranteeing that each color remains unaffected by the others.

The Fourier transform encodes the color information of an object within the high‐frequency components of the Fourier domain. However, the variation amplitude of the adjacent Fourier transform spectra in the high‐frequency regions is significantly reduced compared to those in the low‐frequency regions (Figure S16), which indicates that the light intensity changes in the Fourier transform spectra representing high‐frequency regions are very subtle during Fourier color imaging (Figure S17). To ensure perceptibility, it is necessary for the intrinsic dark current fluctuations of the photodetector to be substantially lower than the minute current variations induced by the diffusely reflected light. Figure 2d illustrates the current fluctuations of the device after continuous operation for 10 000 s under light intensity of 30 µW cm^−2^ conditions. It can be observed that the standard deviation (SD) of the current fluctuations of the CsBi_3_I_10_‐GTC photodetector remains stable after long‐term testing, while the current fluctuations of the CsBi_3_I_10_ photodetector vary a lot under the same condition. This further confirms that the GTC can effectively passivate the CsBi_3_I_10_ and stabilize the device's dark current (Figure S18), enabling reliable recording of the minor signal changes in high‐frequency patterns. To examine the signal amplitude of the photodetectors under light conditions, we further measured the current changes of the CsBi_3_I_10_ photodetector and CsBi_3_I_10_‐GTC photodetector under partial illumination representing high‐frequency regions (Figure 2e). The signal of the CsBi_3_I_10_‐GTC photodetector is effectively captured, whereas the signal of the pristine CsBi_3_I_10_ photodetector is already immersed in the device noise. By comparing the imaging quality under different light intensities, it can be seen that CsBi_3_I_10_‐GTC is more suitable for Fourier single‐pixel color imaging. In low‐light conditions, the imaging quality of the CsBi_3_I_10_‐GTC photodetector exceeds that of traditional SiPMs. (Figure S19).

To reveal the color information encoded within the high‐frequency regions of Fourier space, we captured a 128 × 128 image using the CsBi_3_I_10_‐GTC photodetector, necessitating M = 32 756 measurements for comprehensive sampling. Figure 2f shows the obtained Fourier spectrum at different percentages (S) of the Fourier coefficients (logarithmic modulus) and corresponding reconstructed color images (Figure 2g) as the number of measurements (M) increases. By fully sampling the Rubik's cube (M = 32 756), the reconstructed image has clear contours. However, in modes lacking high‐frequency regions, the reconstructed image only retains the spatial information of the image without the color information. Only when the sampling range covers both low‐frequency and high‐frequency regions can a color image (Figure 2g) be obtained through color restoration algorithms, as demonstrated in the full sampling mode.

### 2.3 Color Fourier Imaging of Wide‐Angle, Weak Light, and High‐Temperature

**Figure 3:**
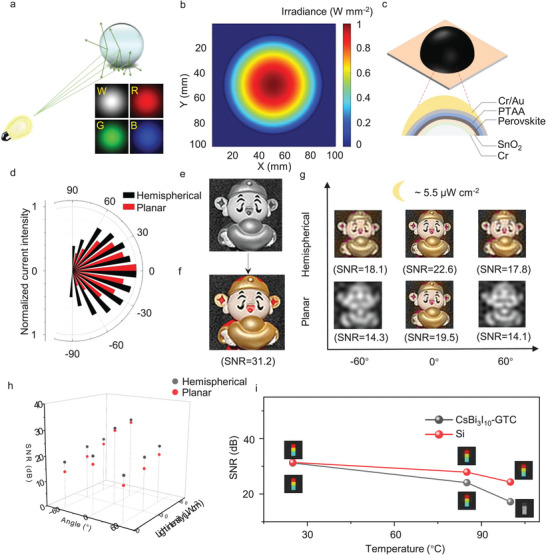
Applicable to filter‐less single‐pixel color imaging in multiple situations. a), The diffuse reflection model of objects illuminated by a projector, and the spatial distribution of light intensity from ideal diffuse reflection of the objects. b), The illumination distribution on the image plane of a Lambertian light field for objects under ideal diffuse reflection conditions. c), The device structure of the hemispherical photodetector. d), The photocurrent variation of hemispherical photodetector and planar photodetector at different positions, as the angle of the photodetector relative to the normal of the object changes (−90°–90°) while the distance remains constant. e), Perform a 2D inverse Fourier transform to reconstruct a high‐resolution (256 × 256) grayscale image from frequency domain data that originally encompassed color information. The photocurrent variation of the hemispherical photodetector and planar photodetector at different positions, as the angle of the photodetector relative to the normal of the object changes (−90°–90°) while the distance remains constant. f), Using a demosaicing algorithm with color restoration, reconstruct a high‐resolution color image. g), Comparison of imaging quality between hemispherical and planar photodetectors at different angles under low‐light conditions (≈5.5 µW cm^−2^). h), Comparison of imaging quality between spherical photodetectors and planar photodetectors under different light intensities and angles. i), Summary of imaging quality between CsBi_3_I_10_‐GTC photodetectors and traditional SiPMs at different temperatures.

As most surfaces are rough, the light they reflect is predominantly scattered diffusely across all directions. We constructed an ideal diffuse reflection model based on a sphere‐shaped subject as an ideal diffuse reflector.^[^ ^28^, ^29^ ^]^ It is expected that the reflected light in all directions gradually attenuates with increasing distance (**Figure** **3**a). To further determine the light intensity distribution of the diffuse light, we established a Lambertian light field for the ideal diffuse reflection model in Figure 3b.^[^ ^30^ ^]^ The theoretical analysis shows that the diffuse reflection light is uniformly scattered in all directions with gradually reduced intensity. Hence, planar photodetectors, like SiPMs, are only capable of capturing the signal from diffuse reflected light at a particular angle, and their spatial degree of freedom is significantly constrained by the inherent limitations of the planar device's receiving area and angle. To receive more diffuse reflected light from different directions to obtain color images and improve image quality, especially under low‐light conditions, we designed a hemispherical photodetector based on spray‐coated CsBi_3_I_10_‐GTC perovskite film (Figure 3c and Figure S20). We compare the current response of this hemispherical photodetector with planar photodetector at different angles (Figure 3d), and the hemispherical geometry shows superior signal feedback due to its effectiveness in receiving light from various angles (Figure S21). Utilizing the hemispherical photodetector, we captured a 256 × 256 resolution color image of a vibrantly colored and structurally unique toy (Figure S22a). Following complete sampling, the reconstructed image exhibits sharp contours and is accompanied by a mosaic template for reference (Figure 3e and Figure S22b). High‐quality color images are recovered from grayscale images using a color restoration algorithm (Figure 3f). To further demonstrate the imaging advantages of the hemispherical photodetector, we apply planar and hemispherical photodetectors for color imaging under different light intensities (5.5, 30, and 51 µW cm^−2^) and angles (0°, 60°, and −60°) (Figure 3g and Figure S23). Imaging quality was quantitatively assessed through SNR calculations. The SNR values, as shown in Figure 3h, confirm the hemispherical photodetector's superior imaging quality over the planar counterpart. The hemispherical photodetector can record color information of images even under weak light conditions (≈5.5 µW cm^−2^) and at extreme angles (60° and −60°).

Imaging is frequently performed in challenging environments, including those with elevated temperatures. Nonetheless, semiconductor thermal noise intensifies markedly with rising temperature, owing to charge carrier transitions between energy bands, ultimately leading to heightened device noise and dark current fluctuations. This phenomenon is common in low bandgap semiconductors such as Si and InGaAs. Therefore the effective signal can be somewhat overshadowed at high temperatures, and results in degraded imaging quality or inability to image. CsBi_3_I_10_‐GTC perovskite exhibits good current stability at high temperatures due to its suitable bandgap and passivated in‐band defects. As the temperature rises from 25 °C to 100 °C, the CsBi_3_I_10_‐GTC photodetector exhibits significantly less current fluctuation compared to SiPMs (Figure S24). To evaluate the color imaging performance of the CsBi_3_I_10_‐GTC photodetector under high‐temperature conditions, we conducted a comparative analysis of the imaging quality between the CsBi_3_I_10_‐GTC photodetector and SiPMs across various temperature ranges (Figure 3i). With increasing temperature, the SNR of color images captured by SiPMs declines markedly (31.4 dB vs 31.2 dB at 25 °C, 27.9 dB vs 24.1 dB at 85 °C, and 24.3 dB vs 17.2 dB at 100 °C), indicating that the CsBi_3_I_10_‐GTC photodetector still can perform color imaging at a high temperature of 100 °C.

## 3 Discussion

In summary, we have successfully proposed a hemispherical single‐pixel photodetector based on spray‐coated lead‐free perovskite film, enabling filter‐less single‐pixel color imaging under complicated environments. The detector boasts exceptional stability, superior low light response capability, and hemispherical device geometry, allowing it to collect more diffuse reflected light and thereby enhancing imaging quality. Additionally, it significantly increases the spatial freedom of the device to accommodate a broader range of application scenarios. This straightforward design not only saves space and cost associated with constructing complex detector arrays but also propels the development of filterless color imaging. Nonetheless, the data acquisition and analysis processes still necessitate substantial computational power, potentially prolonging imaging time and reducing imaging efficiency. Consequently, further model design and algorithm optimization are required to reduce the imaging time.

## 4 Experimental Section

### Film Deposition and Device Fabrication

Thin‐film Preparation: The following was the preparation method for the CsBi_3_I_10_‐GTC perovskite precursor solution. Thoroughly dissolve CsI and BiI_3_ powders with a molar ratio of 1:3 in a mixed solvent of DMF and DMSO with a volume ratio of 9:1, and add different amounts of guanidinium thiocyanate to form the CsBi_3_I_10_‐GTC precursor solution. The prepared precursor solution was uniformly stirred before use. This results in the precursor solution for spray coating. Heat the substrate to ≈110 °C, and add a certain amount of solution to the spray gun. The spray speed was 2 µL s^−1^. After spraying, place the thin film on a heating stage at 125 °C and anneal it for 30 min.

The fabrication process of the hemispherical device was as follows: First, the hemispherical substrate was cleaned thoroughly using water, acetone, and isopropyl alcohol (IPA) in sequence. After cleaning, vacuum evaporation deposition was performed using a chromium (Cr) electrode. During this process, a portion of the hemispherical glass was covered with patterned polyimide (PI) tape to determine the effective working area. Upon completion of drying, the substrate undergoes ultraviolet ozone treatment for 20 min before being transferred to a plasma treatment chamber. Next, the treated hemispherical substrate was fixed onto a stainless steel plate and heated to 100 °C. Meanwhile, the SnO_2_ solution was dissolved in deionized water (DI) at a ratio of 1:12 and evenly sprayed onto the heated substrate using an HD‐180 pneumatic spray gun with a 0.2 mm nozzle. The amount of solution sprayed was adjusted according to the size of the substrate. During the spraying process, the spraying direction was continuously rotated to ensure uniform film thickness of the device. Following the spraying, the Cr/SnO_2_ substrate undergoes thermal annealing at 150 °C for 35 min, and was then transferred to the plasma treatment chamber for 3 min of air plasma treatment. Afterward, the treated substrate was refixed and heated to 110 °C. Subsequently, the perovskite precursor solution was uniformly sprayed onto the processed Cr/SnO_2_ substrate, and nitrogen (N_2_) was used after each spraying to assist in the crystallization of the perovskite surface. Once all spraying work was completed, the entire device undergoes thermal annealing at 125 °C for 30 min. Then, PTAA was dissolved in toluene to prepare a 0.5 mg mL^−1^ solution, which was evenly sprayed onto the heated substrate and thermally annealed at 100 °C for 10 min. Thereafter, the device does not require any additional post‐processing and was directly deposited with 10 nm of Cr and 10 nm of Au via vacuum evaporation.

### The Calculation of Effective Incident Flux Intensity

As shown in Figure S25. After an object was illuminated, it can be regarded as a collection of luminous surface elements (*ds = dxdy*) with a brightness distribution *B(x, y)* and a light intensity reflectivity of *R(x, y)*. The luminous surface elements radiate a flux d*φ* toward a detection unit at a distance of r and with an area of ds_0_, which can be calculated using Equation (1).

(1) $$\begin{array}{c} d\varphi =B\left(x,y\right)d\Omega \cos\theta ds\;=B\left(x,y\right)\frac{ds_{0}\cos\theta^{\prime }}{r^{2}}\cos\theta dx\;dy \end{array}$$

The distance between the detection unit ds0 and the luminous surface element ds was r(x, y, x_0_, y_0_, z). The angle between the connecting line and the normal n of ds was θ2 and the angle with the normal n' of the detection surface element ds_0_ was θ'. $\frac{ds_{0}\cos\theta^{\prime }}{r^{2}}$ was the solid angle of the detection surface element ds_0_ relative to the luminous surface element.

The solid angle relative to the luminous surface element. The luminance of an ideal diffuse surface with illuminance E was given by Equation (2).

(2) $$B\left(x,y\right)=\frac{E\left(x,y\right)}{\pi }\;$$

Considering the reflectance of the surface element ds as R(x, y).

(3) $$B\left(x,y\right)=\frac{E\left(x,y\right)R\left(x,y\right)}{\pi }\;$$

The gray stripe projected onto the object surface with a frequency combination of (fx, fy) and an initial phase of φ_0_ can be calculated in Equation (4).

(4) $$I\left(x,y\right)=a+b\cos\left(2\pi f_{x}x+2\pi f_{y}y+\phi_{0}\right)$$

where a and b represent the background grayscale and contrast of the stripe, respectively. By integrating all the luminous surface sources over the entire object surface, the total radiant flux directly received by the detection surface element ds_0_ can be calculated using Equation (5).

(5) $$\begin{array}{ccc} \phi & = & \int_{S}\;d\phi =\int_{-\infty }^{\infty }\;\int_{-\infty }^{\infty }B\left(x,y\right)\frac{ds_{0}}{r^{2}}cos\theta cos\theta^{\prime }dxdy\\ & = & \int_{-\infty }^{\infty }\;\int_{-\infty }^{\infty }\left[a+bcos\left(2\pi f_{x}x+2\pi f_{y}y+\varphi_{0}\right)\right]\frac{ds_{0}}{r^{2}}cos\theta cos\theta^{\prime }dxdy \end{array}$$

## Conflict of Interest

The authors declare no conflict of interest.

## Author Contributions

H.W. conceived and supervised the project. C.L. fabricated the photodetectors and characterized the performance of the devices. W.L. helped with the analysis of material structure and properties. W.Q. assisted with the single‐pixel point imaging. H.H. contributed to the X‐ray single crystal measurement. J.Z. assisted with the characterization of SEM. All authors analyzed the data. H.W. and C. L. wrote the manuscript, and all the authors commented and reviewed the manuscript.

## Supporting information



Supporting Information

## Data Availability

The data that support the plots within this paper are available from the corresponding author upon request.
