# Supplementary material for: Stable and Lead‐Free Perovskite Hemispherical Photodetector for Vivid Fourier Imaging
Source: Adv Sci (Weinh). 2024 Dec 24;12(7):2414430. doi: 10.1002/advs.202414430 (PMC11831509; doi:10.1002/advs.202414430)
Supplement: Supplementary file 1 — Supporting Information [file ADVS-12-2414430-s001.docx]

**Supplementary Information for**

**Stable and Lead-free Perovskite Hemispherical Photodetector for Vivid Fourier Imaging**

*Chenglong Li, Weijun Li, Wei Qu, Haijing Hu, Jia Zong and Haotong Wei^^[[1]](#footnote-1)^*^*

C. Li, W. Li, W. Qu, H. Hu, J. Zong, Prof. H. Wei

State Key Laboratory of Supramolecular Structure and Materials, College of Chemistry, Jilin University, Changchun, 130012, P.R. China

Prof. H. Wei

Optical Functional Theragnostic Joint Laboratory of Medicine and Chemistry, The First Hospital of Jilin University, Changchun, 130012 P.R. China

*Email: [hweichem@jlu.edu.cn](mailto:hweichem@jlu.edu.cn)

[Color Fourier Imaging by Filterless and Lead-free Perovskite Hemispherical Photodetector 1](#_Toc171620604)

[Supplementary Notes 3](#_Toc171620605)

[Supplementary Methods 4](#_Toc171620606)

[Supplementary Figures and Discussions 12](#_Toc171620607)

[Supplementary References 43](#_Toc171620608)

# Supplementary Notes

### Materials

All chemicals were used without further purification. Bismuth iodide (BiI_3_, 99.99% powder), cesium iodide (CsI, 99.99% powder), poly[(4-phenyl)(2,4,6-trimethylphenyl)amine] (PTAA, Mw: 1000-10000 by GPC) were purchased from Xi'an p-OLED. Anhydrous N,N-Dimethylformamide (DMF, 99.9%), N,N-Dimethylsulfoxide (DMSO, 99.9%), anhydrous methanol (MeOH, 99.99%), and guanidinium thiocyanate (GTC) were purchased from Energy Chemical. SnO_2_ colloid precursor (tin (IV) oxide, 15% in H_2_O colloidal dispersion) was obtained from Alfa.

## General Information

UV-visible absorption spectra were obtained using a Shimadzu 3600 UV-visible-NIR spectrophotometer. Photoluminescence spectra were acquired using Omni-λ3007i. XRD data were collected using a PANalytical B.V.-Empyream Diffractometer with Cu Kα radiation. SEM cross-section images were captured using a Hitachi Cold Field Emission SEM equipped with Bruker X-ray. Fourier Transform Infrared Spectroscopy (FTIR) was obtained through a Thermofisher Scientific Nicolet iS10 FFT Infrared Spectrometer connected to a Smart iTX. The response time was recorded by an MDO3000 oscilloscope under 650 nm LED light triggered by a RIGOL DG1000 function generator. The Noise Equivalent Power (NEP) was measured by a Keysight E35670A FFT dynamic signal analyzer under laser light triggered by the RIGOL DG1000 function generator. Current and voltage correlation tests were conducted using a Keithley 2400 SourceMeter.

# Supplementary Methods

## Parameter calculation of photoelectric detector

Responsiveness (R) is defined as the ratio of the photocurrent to the irradiance of light in a device, which is used to describe the magnitude of the current generated by illumination.

$R=\frac{J_{ph}}{L_{light}}$ (1) where Jph represents the photocurrent density, and Llight represents the incident light irradiance.

Specific detectivity (D*) is another figure-of-merit of a photodetector, which can be calculated by the following equation:

$D^{*}=\frac{\sqrt{AB}}{\mathrm{NEP}}$ (2)

where A is the active device area, the active device area is 0.1 cm^2^; B is the electrical bandwidth of the noise measurement. To achieve standardization, we set the electrical bandwidth to 1 Hz, which reflects the detection capability per unit bandwidth.

NEP is the noise equivalent power determined by

$\mathrm{NEP}=\frac{i_{\text{noise }}}{R}$ (3)

where R is responsivity and *i_noise_* is the noise current determined by the FFT measurement.

Linear dynamic range (LDR) is used to describe the linear response capability of a detector to different light intensity ranges. In this linear region, the responsivity value remains constant. That is to say, in logarithmic representation, the photocurrent should be 1. In principle, the lower limit of LDR is the noise current, which highly depends on the bias voltage. The upper limit of LDR is the competition between charge extraction and bimolecular recombination. For traditional photodiodes, applying a reverse bias voltage can enhance charge extraction to improve responsivity and linearity. LDR is usually expressed in logarithmic form.

$LDR=20\log\frac{J_{\text{upper }}-J_{\text{dark }}}{J_{\text{lower }}-J_{\text{dark }}}$ (4)

where J_upper_ is the current density generated by the highest light intensity that the device can respond to linearly, while J_lower_ is the current density generated by the lowest light intensity that the device can respond to linearly. J_dark_ is the dark current of the device.

## Calculation of trap state density (tDOS)

The demarcation energy *Eω* correlates with the applied frequency by the following expression:

$E_{\omega}=k_{B}Tln(\frac{\omega_{0}}{\omega})$ (5)

where *ω_0_* is the attempt-to-escape frequency, *ω* is the applied angular frequency, *k_B_* is the Boltzmann constant, and *T* is the absolute temperature. The distribution of trap density of states *N_T_* can be calculated by:

$N_{T}\left( E_{\omega} \right)=-\frac{V_{bi}}{qW}\frac{dC}{d\omega}\frac{\omega}{k_{B}T}$ (6)

where *V_bi_* is the build-in potential, *W* is the width of the depletion region, and the thickness of active layer is employed as *W*. *q* is the elementary charge.

## Four-step phase-shifting method

Four-step phase shifting technology can be used for the 3D reconstruction of structured light in traditional imaging. Its basic principle is to project structured light of a specific pattern through projection equipment, and analyze and calculate it to obtain the pixel correspondence between the projection equipment and the camera. A three-dimensional reconstruction of the target object can be carried out through preset system parameters^[1]^. The four-step phase shift phase demodulation uses four sets of projected structured light to calculate phase for each point in the camera, corresponding to the pixels of the projection equipment. The principle of four-step phase shifting is as follows:

The sinusoidal grating light intensity projected by the projector can be expressed as Eq. (7). $I\left( x,y,\phi\right)=I_{0}\left( x,y \right)+A\left( x,y \right)cos\left[ \psi\left( x,y \right)+\phi\right]$ (7)

where I_0_(x, y) represents the background intensity of the image, A(x, y) represents the modulation amplitude, ψ(x, y) represents the corresponding phase value at (x, y), and ϕ represents the phase shift value^[2]^. The phase shift values are 0, π/2, π, 3π/2, and the light intensity expression is given by Eq. (8).

$\begin{matrix} I_{1}\left( x,y \right)=I_{0}\left( x,y \right)+A\left( x,y \right)cos\left[ \psi\left( x,y \right)+0 \right] \\ I_{2}\left( x,y \right)=I_{0}\left( x,y \right)+A\left( x,y \right)cos\left[ \psi\left( x,y \right)+\frac{\pi}{2} \right] \\ I_{3}\left( x,y \right)=I_{0}\left( x,y \right)+A\left( x,y \right)cos\left[ \psi\left( x,y \right)+\pi\right] \\ I_{4}\left( x,y \right)=I_{0}\left( x,y \right)+A\left( x,y \right)cos\left[ \psi\left( x,y \right)+\frac{3\pi}{2} \right] \end{matrix}$ (8)

From the above equation, the phase value at (x, y) can be calculated as shown in Eq. (9).

$\psi\left( x,y \right)=arctan\left( \frac{I_{4}-I_{2}}{I_{1}-I_{3}} \right)$ (9)

## Continuous Fourier Transform

Continuous Fourier Transform decomposes a function into a continuous spectrum. For any periodic function, it can be represented as a sum of sine (cosine) waves of different frequencies^[3]^. No matter how complex the function is, as long as it is periodic and satisfies certain mathematical conditions, it can be expressed as a "sum". Any continuous signal can be linearly combined from a set of Fourier bases (Eq. (10-11)).

$G\left( f_{x},f_{y} \right)=\int_{-\infty}^{+\infty}\int_{-\infty}^{+\infty}g\left( x,y \right)exp\left[ -j\cdot2\pi\left( f_{x}x+f_{y}y \right) \right]dx\sim dy$ (10)

$g\left( x,y \right)=\int_{-\infty}^{+\infty}\int_{-\infty}^{+\infty}G\left( f_{x},f_{y} \right)exp\left[ j\cdot2\pi\left( f_{x}x+f_{y}y \right) \right]df_{x}\sim df_{y}$ (11)

where j is the imaginary unit, x, and y are plane coordinates, f_x_ is the spatial frequency corresponding to the x-direction, f_y_ is the spatial frequency corresponding to the y-direction, n(x, y) represents a two-dimensional image, and G($f_{x},f_{y}$) is the Fourier transform or spectrum of g(x, y).

## Fourier Single-Pixel Imaging

Conventional single-pixel imaging reconstructs the image of a target object by analyzing the intensity correlation before signal acquisition. In contrast, Fourier single-pixel imaging employs a different approach: it first captures the reflected signals from the object illuminated by structured light of different spatial frequencies, and then utilizes a phase-shifting algorithm to obtain a complete spectrum containing all Fourier coefficients of the object. Finally, the reconstructed image of the object is obtained through inverse Fourier transformation of this spectrum. In terms of light source modulation, traditional single-pixel imaging generates a series of random speckle patterns through a computer, which are then sent to a spatial light modulator to produce structured light that follows a specific distribution. This structured light is subsequently projected onto the target object. Fourier single-pixel imaging, on the other hand, begins by generating Fourier basis patterns containing specific frequency and phase information through a computer. It then controls a projector to generate corresponding structured light (spatial frequency domain fringes) to illuminate the object. Compared to traditional methods, Fourier single-pixel imaging exhibits significant advantages in light source modulation and object reconstruction techniques, thereby achieving notable improvements in both imaging speed and quality.

The Fourier single-pixel imaging principle involves projecting Fourier basis patterns, known as spatial frequency domain fringes and described by Eq. (12), onto the object to capture its image.

$P_{\phi}\left( x,y;f_{x},f_{y} \right)=a+bcos\left( 2\pi f_{x}x+2\pi f_{y}y+\phi\right)$ (12)

where a denotes the DC component of the illumination pattern, b represents the fringe contrast, and ϕ signifies the initial phase. If the four-step phase-shifting algorithm is adopted, the Fourier basis patterns can be calculated using Eq. (13).

$\left\{ \begin{matrix} P_{1}\left( x,y;f_{x},f_{y} \right)=a+bcos\left( 2\pi f_{x}x+2\pi f_{y}y+0 \right) \\ P_{2}\left( x,y;f_{x},f_{y} \right)=a+bcos\left( 2\pi f_{x}x+2\pi f_{y}y+\pi/2 \right) \\ P_{3}\left( x,y;f_{x},f_{y} \right)=a+bcos\left( 2\pi f_{x}x+2\pi f_{y}y+\pi\right) \\ P_{4}\left( x,y;f_{x},f_{y} \right)=a+bcos\left( 2\pi f_{x}x+2\pi f_{y}y+3\pi/2 \right) \end{matrix} \right.$ (13)

The total reflected light intensity after spatial frequency domain fringes illuminate the target object can be expressed as Eq.(14):

$I(f_{x},f_{y})=\iint_{s} O(x,y)P_{\varphi}(x,y;f_{x},f_{y})dxdy$ (14)

where s represents the illuminated area of the basis pattern, and O(x, y) denotes the spatial distribution of the target object. Considering the potential impact of ambient background light noise on detection during the experiment, the total reflected light intensity from the target object, as measured by the single-pixel detector, can be calculated using the following Eq. (15):

$I(f_{x},f_{y})=\iint_{s} O(x,y)P_{\varphi}(x,y;f_{x},f_{y})dxdy$ (15)

where s represents the illuminated area of the basis pattern, and O(x, y) denotes the spatial distribution of the target object. Considering the possible influence of ambient background noise during the experiment, the total reflected light intensity from the target object, measured by the single-pixel detector, can be calculated using the following Eq. (16):

$B_{\varphi}(f_{x},f_{y})=B_{n}+\gamma\cdot I(f_{x},f_{y})$ (16)

where $B_{n}$ represents the ambient background light noise intensity, and $\gamma$ denotes a parameter related to the detector's gain. By substituting Eq. (8) and Eq. (9) into Eq. (10), it can be obtained that when using the four-step phase-shifting algorithm, the total reflected light intensity measured by the single-pixel detector can be calculated using Eq. (17). $\begin{aligned} B_{1}(f_{x},f_{y})=B_{n}+a\gamma\iint_{8} O(x,y)dx\text{ }dy+b\gamma\iint_{8} O(x,y)\cos(2\pi f_{x}x+2\pi f_{y}y+0)dx\text{ }dy \\ B_{2}\left( f_{x},f_{y} \right)=B_{n}+a\gamma\iint_{8} O(x,y)dx\text{ }dy+b\gamma\iint_{8} O(x,y)\cos(2\pi f_{x}x+2\pi f_{y}y+\pi/2)dx\text{ }dy \\ B_{3}(f_{x},f_{y})=B_{n}+a\gamma\iint_{8} O(x,y)dx\text{ }dy+b\gamma\iint_{8} O(x,y)\cos(2\pi f_{x}x+2\pi f_{y}y+\pi)dx\text{ }dy \\ B_{4}(f_{x},f_{y})=B_{n}+a\gamma\iint_{8} O(x,y)dx\text{ }dy+b\gamma\iint_{8} O(x,y)\cos(2\pi f_{x}x+2\pi f_{y}y+3\pi/2)dx\text{ }dy \end{aligned}$ (17)

Based on the four-step phase-shifting algorithm, the spectrum of the object, namely the Fourier coefficients, can be obtained through the following Eq. (18):

$\begin{matrix} C(f_{x},f_{y}) & =[B_{1}(f_{x},f_{y})-B_{3}(f_{x},f_{y})]+j\cdot[B_{2}(f_{x},f_{y})-B_{4}(f_{x},f_{y})] \\ & =2b\gamma\cdot F\{O(x,y)\} \end{matrix}$ (18)

where F{} represents the Fourier transform. By projecting structural light fringes of different spatial frequencies onto the target object, a complete spectrum containing all Fourier coefficients of the object can be obtained. Then, the image reconstruction of the target object can be achieved by utilizing the inverse Fourier transform formula (Eq. (19)).

$O(x,y)=\frac{1}{2b\gamma}\cdot F^{-1}\{C(f_{x},f_{y})$ (19)

where F^-1^{} represents the inverse Fourier transform.

## Fourier single-pixel color imaging

The imaging process of Fourier single-pixel color imaging is the same as the Fourier single-pixel imaging process mentioned earlier. However, the gray Fourier basis needs to be adjusted using a Bayer template to generate color Fourier basis patterns^[4]^. The gray-scale pattern is modulated by a Bayer template, where the relationship between the gray-scale pattern and the Bayer filter template is multiplicative^[5]^. That is, the final illumination pattern obtained is the result of the point-by-point multiplication of the gray-scale pattern and the Bayer template in the spatial domain, as shown in Eq. (20).

$P_{\phi}(x,y,\phi,c)=\{\begin{aligned} M_{R}\cdot P_{\phi}(x,y,\phi),c=R \\ M_{G}\cdot P_{\phi}(x,y,\phi),c=G \\ M_{B}\cdot P_{\phi}(x,y,\phi),c=B \end{aligned}$ (20)

where c represents color, by combining the above three monochromatic Fourier basis patterns into a three-layer matrix, we obtain a series of Fourier color basis patterns with different frequencies and phases.

# Supplementary Figures and Discussions

## Establishment of Fourier single-pixel imaging model

The fundamental principle of Fourier single-pixel imaging lies in obtaining the Fourier transform spectrum of the object image through spatial light modulation, and then reconstructing the object image by performing an inverse Fourier transform on the Fourier spectrum A 2D image consisting of I×J pixels can be converted into a spectral space with corresponding I×J complex Fourier coefficients via Fourier spectroscopy. Afterward, by utilizing the inverse Fourier transform, these complex Fourier coefficients can be reconverted into the original 2D image (Figure S1).


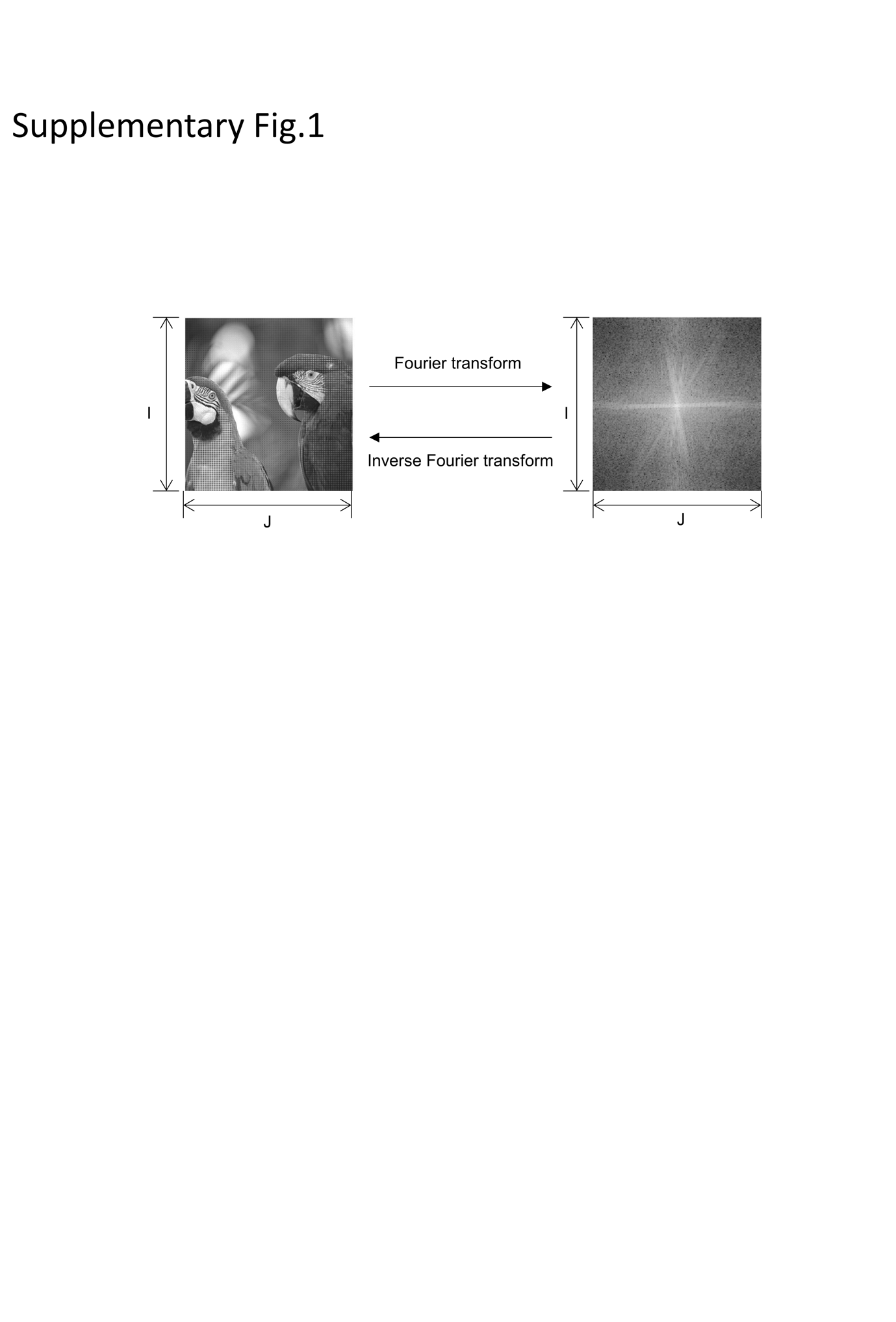


**Figure S1. Method for obtaining Fourier coefficients.** The left image is the original photograph. The right image shows the logarithmic modulus of the Fourier spectral coefficients.

Fourier single-pixel imaging is primarily achieved through the four-step phase-shifting method mentioned earlier. This method involves projecting four Fourier basis patterns with the same spatial frequency but different initial phases: 0, π/2, π, and 3π/2, to obtain the corresponding Fourier coefficient (Figure S2).


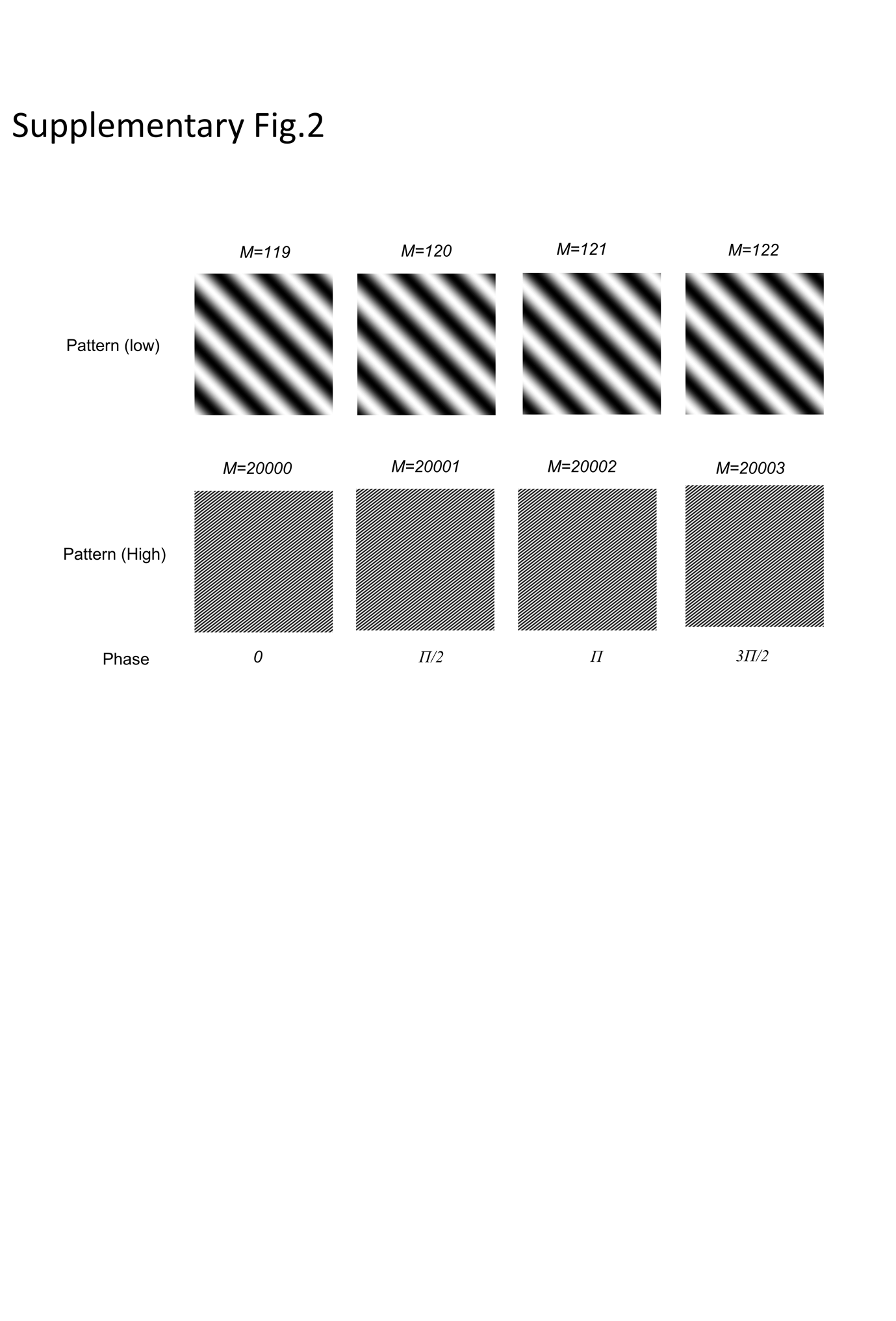


**Figure S2. Fourier spectrum under different phases.** Four Fourier basis patterns with the same spatial frequency (low and high frequencies).

Figure S3a illustrates the development of RGB mode in imaging technology based on the principle of human eye perception. Because the Fourier base pattern of grayscale is used for illumination, the reconstructed object pattern is also grayscale (Figure S3b). Based on the theory of single-pixel monochrome imaging, color imaging can be achieved through color separation illumination combined with time-division signal acquisition imaging. For each frequency of projected fringe pattern, monochromatic illumination is performed in the order of red, green, and blue (Figure S3c). After the completion of spectrum reconstruction, three monochromatic images are obtained, which are then combined to produce a color image (Figure S3d).


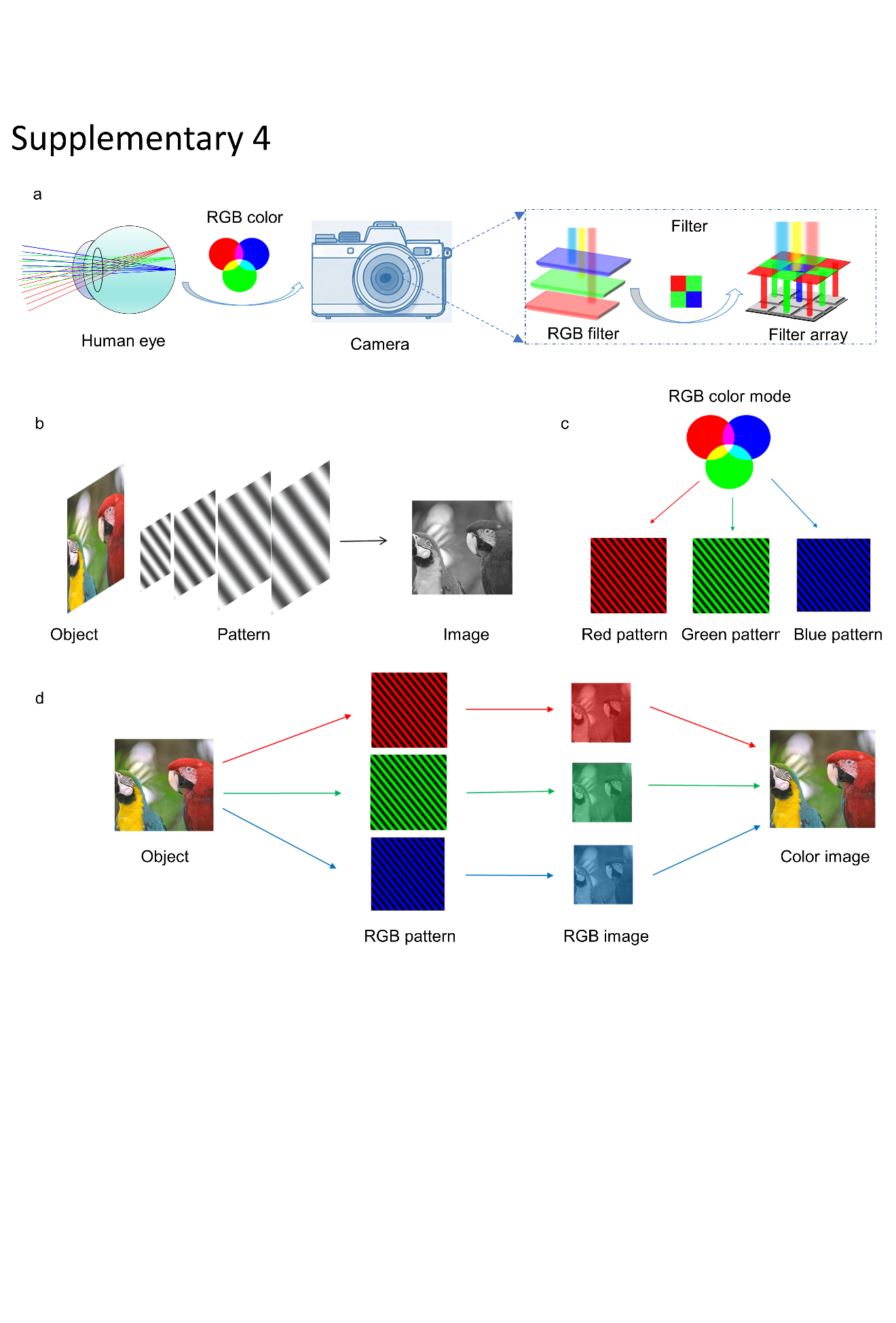


**Figure S3. Schematic diagram of single-pixel color imaging using RGB pattern. a,** The development of RGB mode in imaging technology. **b,** The formation process of Fourier single-pixel imaging grayscale image. **c,** Schematic diagram of RGB pattern. **d,** Color single-pixel imaging model using three RGB patterns.

## The characterization of CsBi_3_I_10_ (w GTC) film

Figure S4a shows the structural models of CsBi_3_I_10_ perovskite crystals from different angles. GTC is an organic compound with a molecular formula of C_2_H_6_N_4_S Structurally speaking, it consists of carbon, hydrogen, nitrogen, and sulfur atoms, forming a unique molecular structure (Figure S4b). In this molecule, carbon atoms are connected to hydrogen and nitrogen atoms, constituting a stable framework. Meanwhile, a thiocyanate radical is formed between sulfur and nitrogen atoms^[6-7]^. Figure S4c is a molecular structure model showing the interaction between perovskite crystals and GTC. GA^+^ cations can effectively passivate the cation vacancy defects and Bi-I antisite defects on the bottom surface of perovskite thin films through hydrogen bonding and electrostatic interactions between their amino groups. Secondly, due to its similar chemical behavior to I^-^ (the ionic radius of SCN^-^ is 217 pm, which is close to that of I^-^ at 220 pm), the lone pair of electrons in the linear structure of SCN^-^ can coordinate with Bi^3+^, thereby regulating the nucleation and growth process of CsBi_3_I_10_.


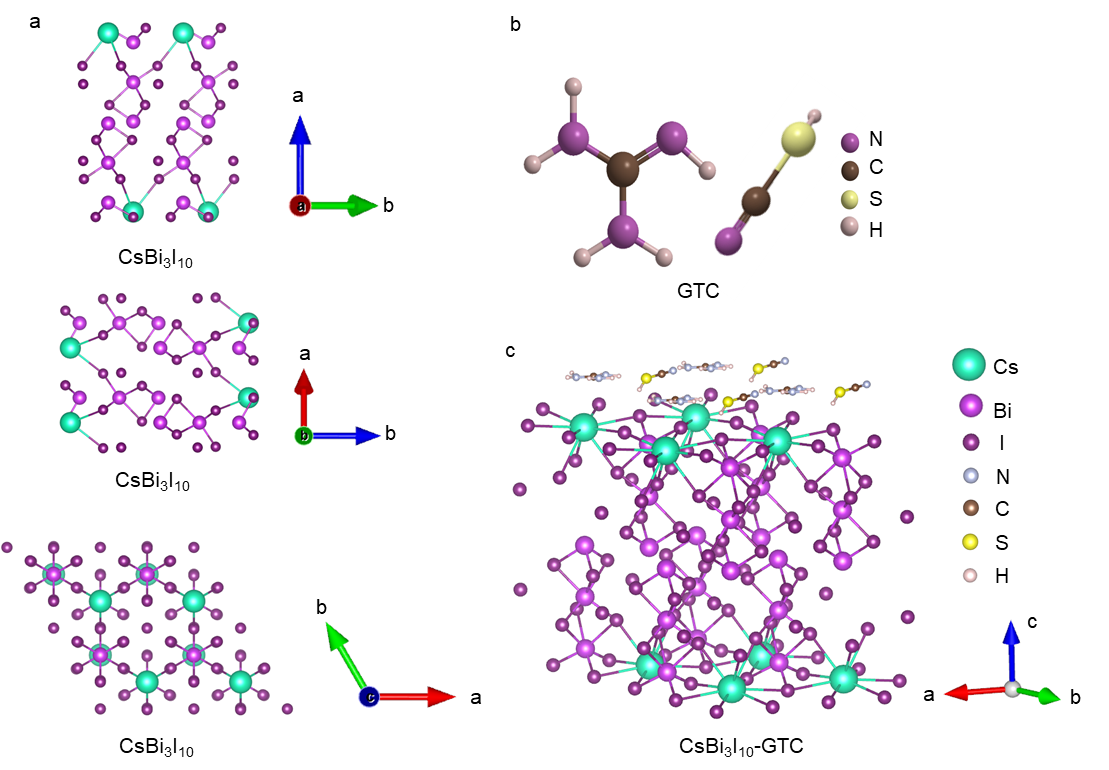


**Figure S4. Crystal structure model. a,** CsBi_3_I_10_ perovskite crystal structure. **b,** Ball-and-stick model of GTC molecular formula. **c,** Interaction model between CsBi_3_I_10_ perovskite and GTC.

To verify the presence of trace amounts of SCN^-^ in the grown perovskite film, we used Fe^3+^ for a color reaction. After mixing FeCl_3_ with CsBi_3_I_10_-GTC perovskite powder in N,N'-Dimethylformamide (DMF) solution, the color immediately changed from light yellow to blood red (Figure S5), while this phenomenon was not observed in the CsBi_3_I_10_ solution. This color change indicates the presence of trace amounts of SCN^-^ in the grown perovskite film^[8]^.


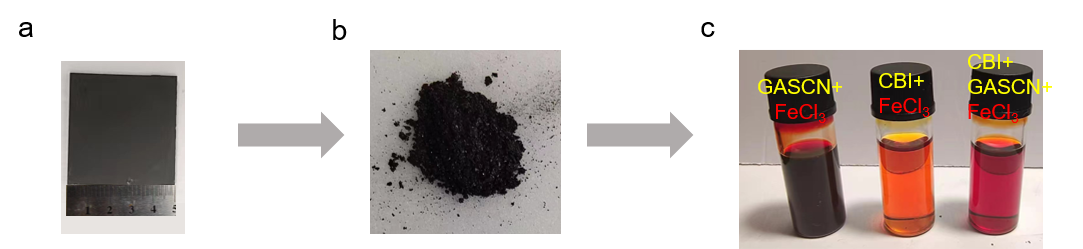


**Figure S5. Chromogenic reaction.** **a,** the state of a large-area CsBi_3_I_10_-GTC perovskite film (5×5 cm²) after thermal annealing at 125 ℃ for 30 minutes. **b,** the powder scraped from the large-area perovskite film. **c,** photos of chromogenic reactions of GTC, CsBi_3_I_10_, and CsBi_3_I_10_-GTC with Fecl_3_.


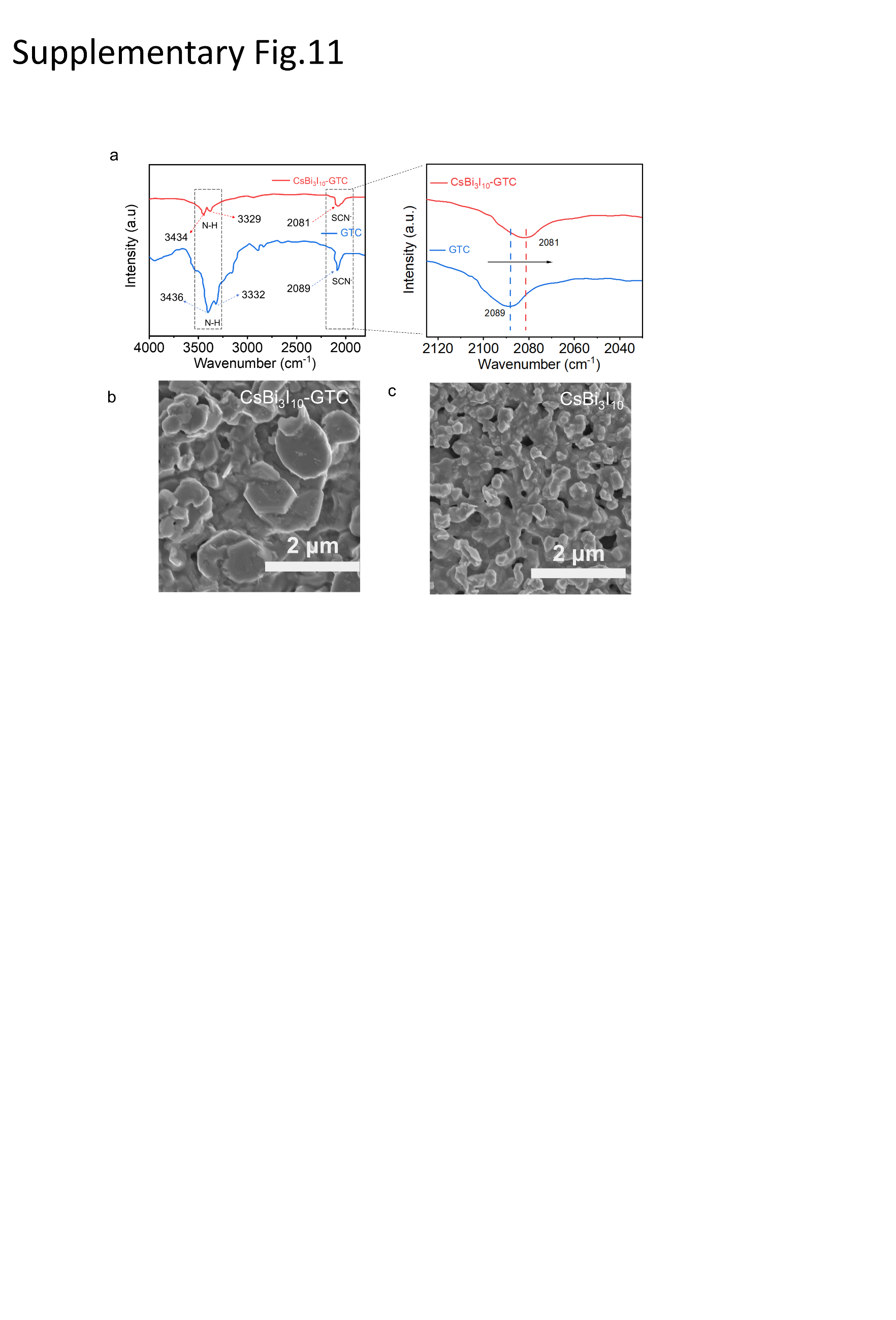


**Figure S6. Structure and characteristics of CsBi_3_I_10_ and CsBi_3_I_10_-GTC membranes. a,** FTIR spectra of GTC powder and CsBi_3_I_10_-GTC mixture in the amplitude range. **b,** SEM image of CsBi_3_I_10_-GTC films. **c,** SEM image of CsBi_3_I_10_ films.

**Spectral information of projectors**

To quantify the spectral band of detection, we utilized a fiber spectrometer to measure the spectral output of the digital projector while displaying pure red, green, and blue patterns. The spectra obtained are presented in Figure S7. For the red channel, the central wavelength is 625 nm with a full wave at half maximum (FWHM) of 19.4 nm. Regarding the green channel, the central wavelength is 545 nm and the FWHM is 79.8 nm. Lastly, for the blue channel, the central wavelength stands at 445 nm and the FWHM is 23.8 nm.


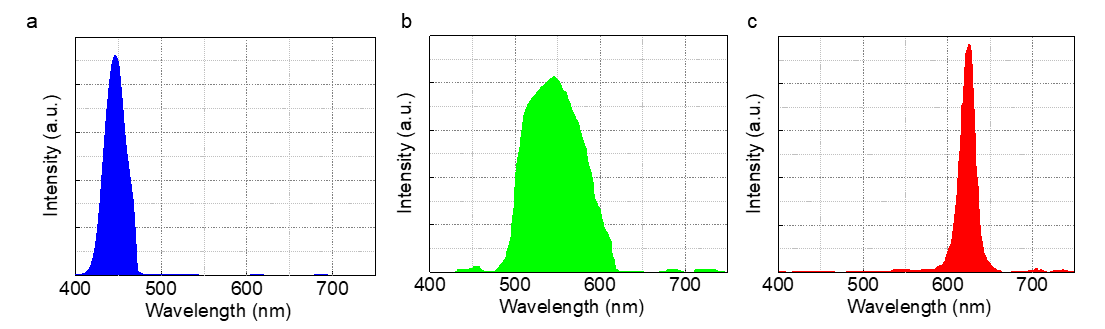


**Figure S7. Intensity comparison of different colors of light projected by a projector.** **a,** Blue. **b,** Green. **c,** Red.

## Performance characterization of photodetectors

The perovskite precursor solution is sprayed onto the substrate surface, and a dry nitrogen purge is required after each deposition of the film until most of the solvent is volatilized and removed. During the spraying process, the spraying direction should be continuously rotated to ensure the uniformity of the film thickness of the device (Figure S8a). The schematic diagram of the device structure is Figure S8b (ITO/SnO_2_/CsBi_3_I_10_/PTAA/Cr).


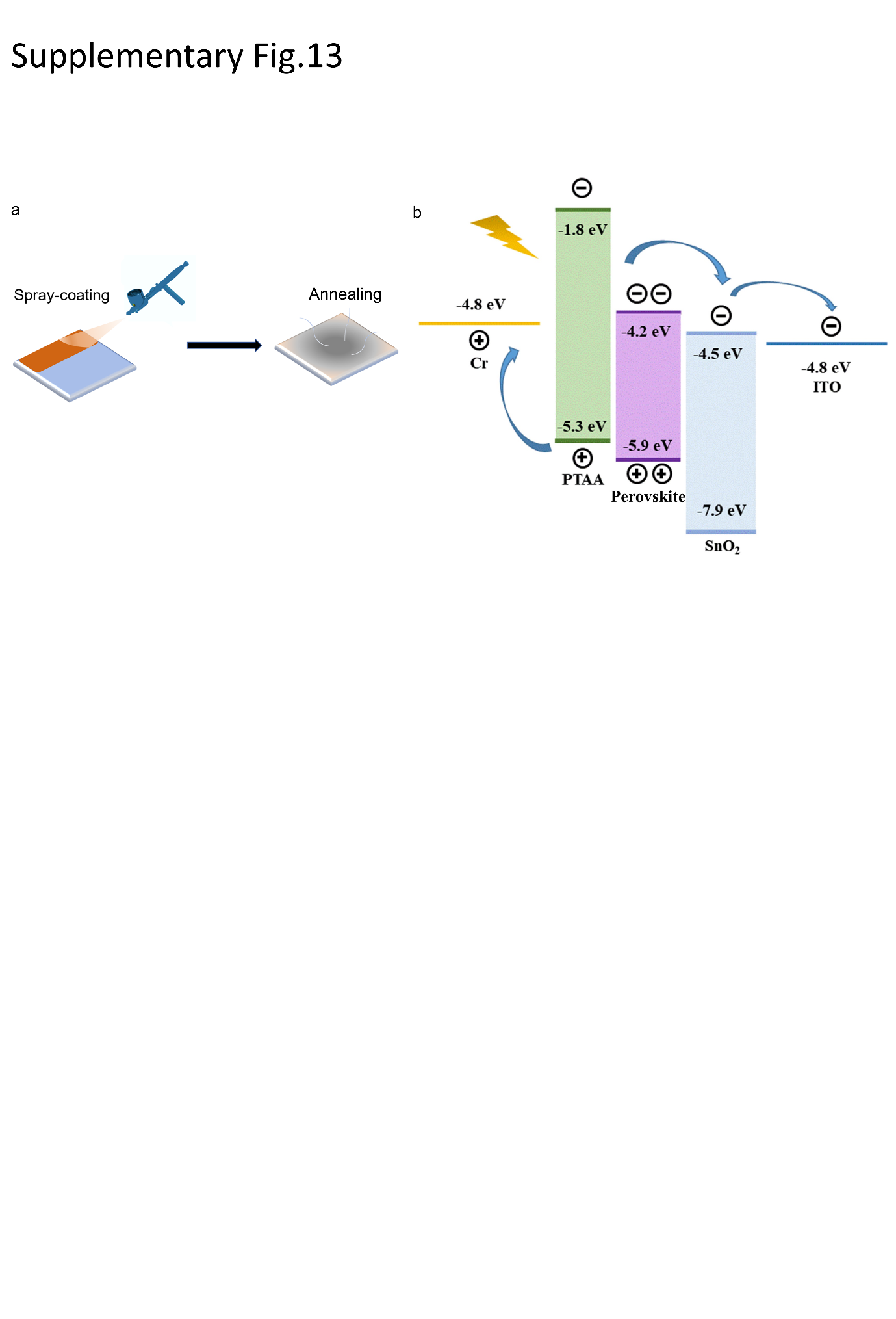


**Figure S8. Device Structure and Spraying Process. a,** An orange film is obtained by spraying the precursor solution, and the film turns black after annealing. **b,** Structure diagram of perovskite photodetector.

Photodetector devices made from CsBi_3_I_10_ films, both doped and undoped with CsBi_3_I_10_, underwent photoelectric testing, and the results are presented in Figure S9a. Under solar illumination with an intensity of 100 mW cm^-2^, the photocurrent of the CsBi_3_I_10_-GTC device shows a significant improvement compared to the CsBi_3_I_10_ device. Figure S9b illustrates the photocurrent (I-P) curve related to light intensity for the CsBi_3_I_10_-GTC photodetector, and its corresponding linear dynamic range (LDR) is calculated to be 98 dB. This enhancement is attributed to the introduction of GTC into the CsBi_3_I_10_ film. Figure S9c displays the normalized spectral responsivity of the CsBi_3_I_10_-GTC photodetector. The device exhibits significant photoresponse within the wavelength range of 340 to 700 nm, which aligns with the absorption curve of the CsBi_3_I_10_-GTC film. The CsBi_3_I_10_-GTC device demonstrates the highest photoresponsivity at 650 nm. The response speed of a photodetector is another crucial parameter for evaluating its responsiveness to optical signals. The rise time (t_r_) and decay time (t_d_) are defined as the time required for the photocurrent to increase from 10% to 90% of its maximum value, and to decrease from 90% to 10% of its maximum value, respectively. Under illumination with a wavelength of 650 nm, the estimated tr and td of the device are 13 µs and 10 µs, respectively (Figure S9d).

**
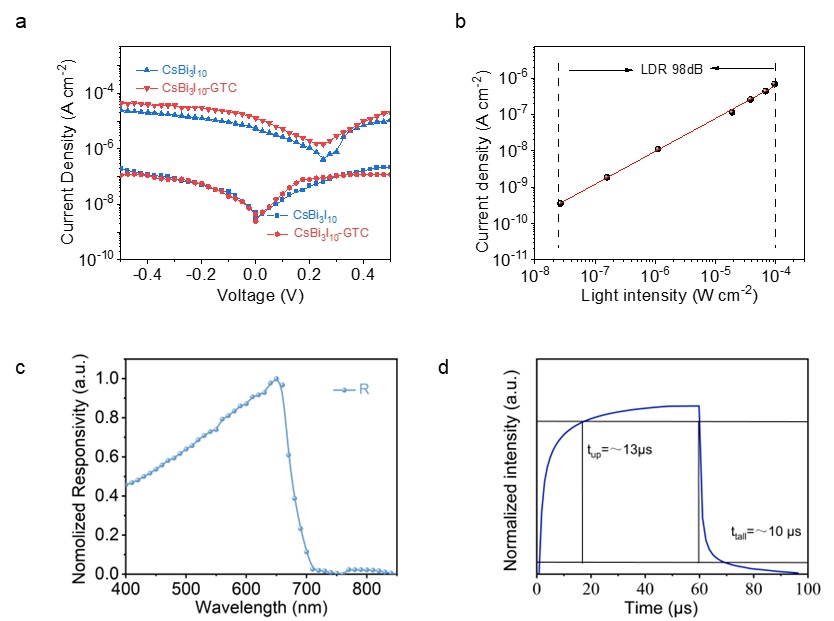
**

**Figure S9. Performance characterization of photodetectors. a,** Spectral response of the CsBi_3_I_10_-GTC photodetector. **b,** I-P curve of the CsBi_3_I_10_-GTC photodetector, along with the corresponding LDR. **d,** Time-dependent voltage curve of the CsBi_3_I_10_-GTC photodetector to measure response time.

## Stability of CsBi_3_I_10_-GTC and CsBi_3_I_10_ photodetectors

The addition of GTC not only enhances the photoelectric performance of CsBi_3_I_10_ but also boosts its stability, which can be categorized into long-term stability and photo-stability. As illustrated in the absorption spectrum of Figure S10a, the CsBi_3_I_10_ perovskite film exhibits excellent long-term stability, enduring 30 days of aging at RH 40%～85% without any decomposition. To assess water stability, both the original CsBi_3_I_10_ and CsBi_3_I_10_-GTC films were fully soaked at room temperature, as demonstrated in Figure S10b. Notably, the black CsBi_3_I_10_ film turned yellow after just 60 s, indicating a rapid reaction between CsBi_3_I_10_ crystals and water molecules. In contrast, the CsBi_3_I_10_-GTC film maintained its black color even after 360 s of soaking under the same conditions. These findings underscore the effectiveness of the GTC additive in mitigating degradation mechanisms and enhancing the water stability of the CsBi_3_I_10_ film. Figure S10c illustrates a typical I-t curve for the CsBi3I10 photodetector under the same light intensity. There is a significant decrease in photocurrent over 10,000 s.

Figure S10d presents the XRD patterns and film photographs of the CsBi_3_I_10_-GTC film at various temperatures. At 150 °C, the CsBi_3_I_10_-GTC film retains its black color, and its XRD characteristic peak (003) remains prominent, highlighting its excellent high-temperature stability. As the temperature gradually increases, the film begins to turn orange, and its XRD characteristic peak shifts to being dominated by (006), indicating a gradual transformation towards Cs_3_Bi_2_I_9_. The conversion of CsBi_3_I_10_ is almost complete at 280 °C.

Figure S10e depicts the I-t curves of the CsBi_3_I_10_ photodetector at 100 °C under a light intensity of 30 μW cm². Notably, the photocurrent of the CsBi_3_I_10_-GTC photodetector remains relatively stable over one hour, further validating the excellent high-temperature stability of the CsBi_3_I_10_-GTC device.


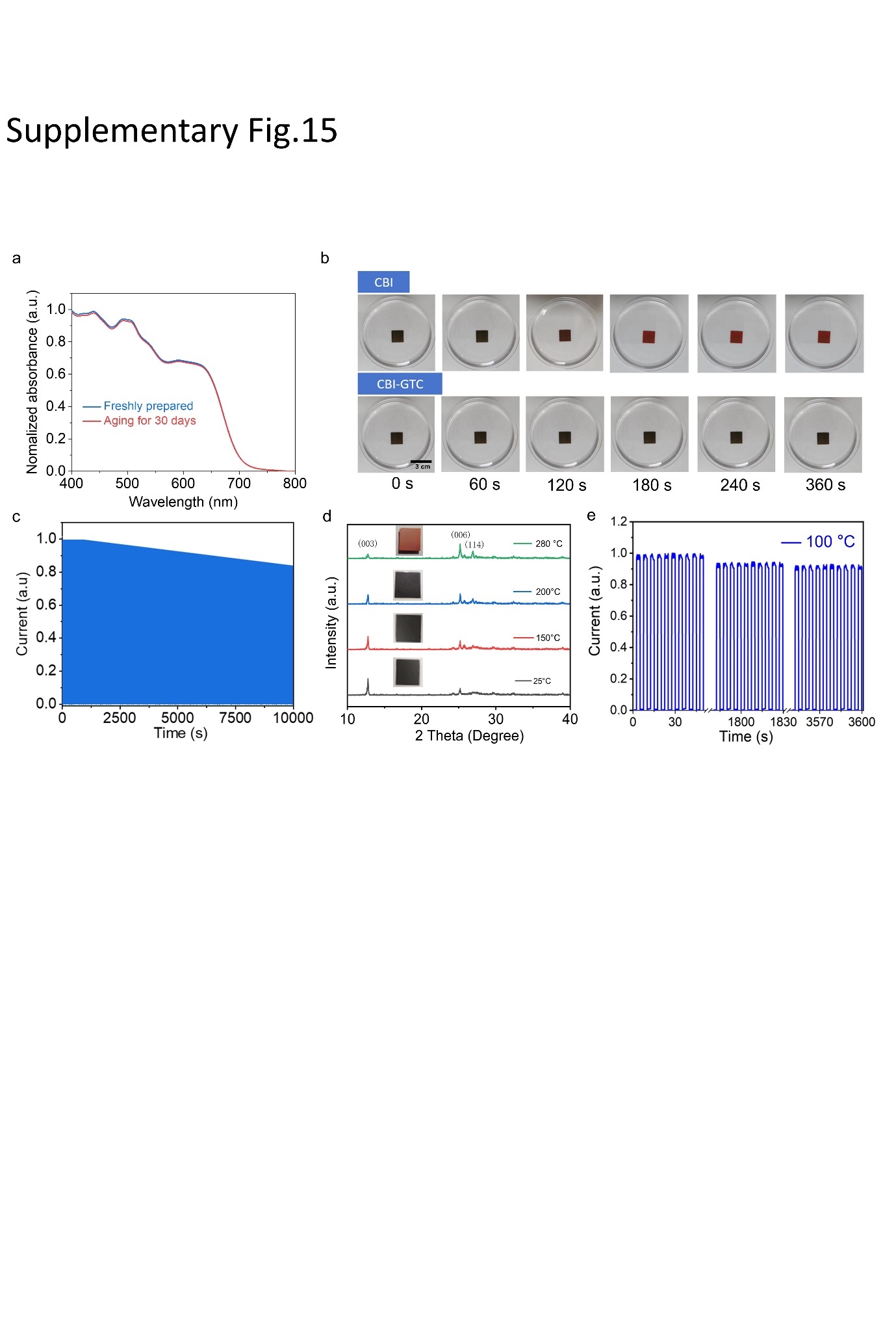


**Figure S10. The stability of the CsBi_3_I_10_-GTC film fabricated by spray-coating. a,** The absorbance of the CsBi_3_I_10_-GTC freshly prepared and after aging for 30 days at ambient (R.H. 40% ~ 85%). **b,** Images of CsBi_3_I_10_ and CsBi_3_I_10_-GTC films which were immersed in water for 360 s. **c,** The I-t curve of the CsBi_3_I_10_ device under 10,000 s optical switching cycle. **d,** XRD patterns of CsBi_3_I_10_-GTC films at different temperatures (the insets are photos of the films taken at various temperatures). **e,** Time-dependent photoresponse curves of CsBi_3_I_10_ photodetectors at 100 °C for 3600s.

Since spatial information is primarily recorded in the low-frequency region of the Fourier transform, we can obtain high-quality grayscale images with just 30% sampling (Figure S11).


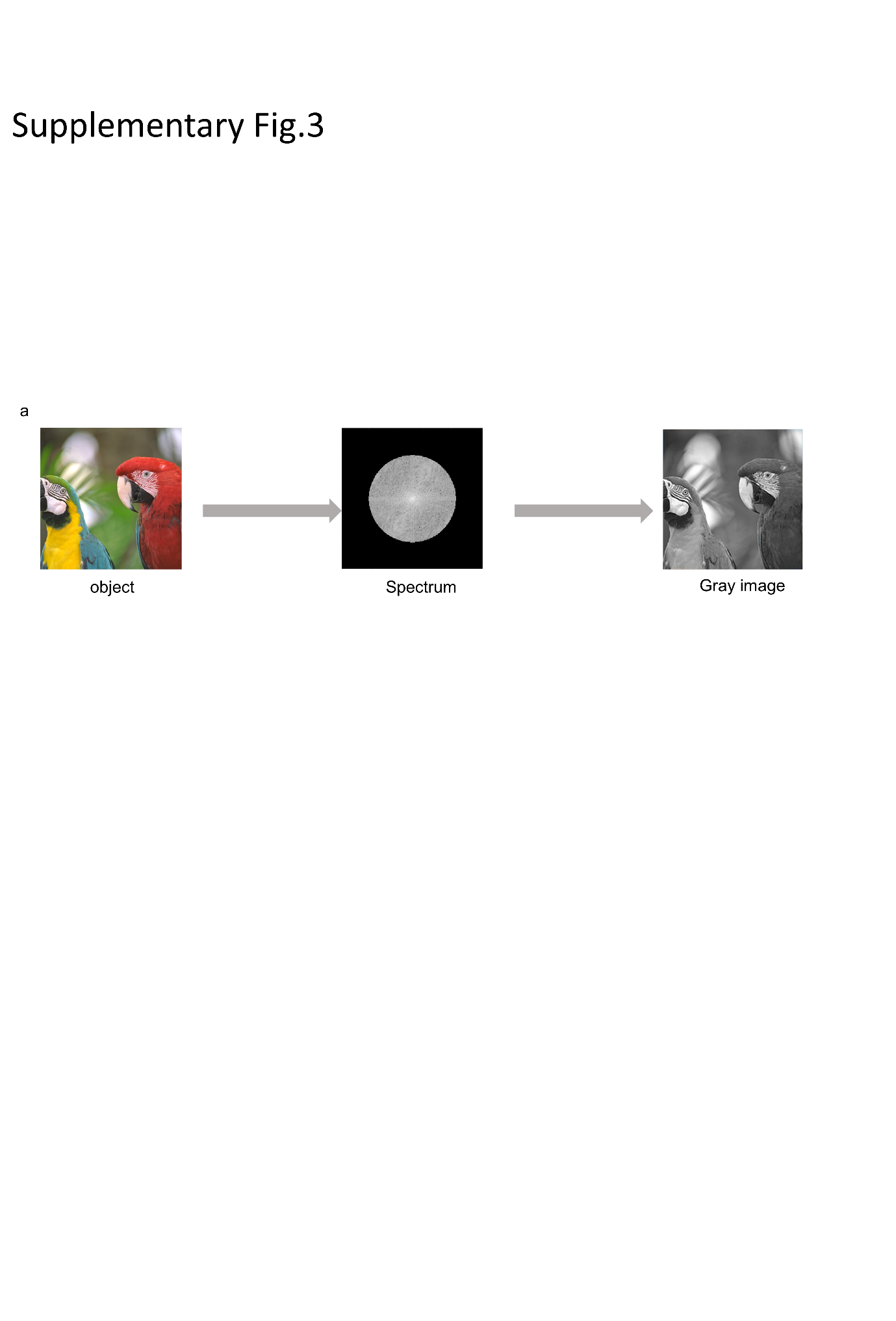


**Figure S11. Fourier single-pixel imaging at low frequencies.** The spectrum obtained by sampling along a circular path. The figure shown represents the spectrum acquired at a sampling rate of 30%.

The four-step phase-shifting sinusoidal color pattern projected by the projector can be regarded as the light source. A hemispherical photodetector is used to collect the current signals induced by diffuse reflected light under different color patterns. The collected current signals contain spatial and color information of the two-dimensional image, which can be reconstructed using the inverse Fourier transform algorithm. The color information contained in the reconstructed grayscale 2D image is encoded in a mosaic template. A de-mosaic algorithm for color restoration is used to reconstruct the color image of the object from the grayscale image (Figure S12).


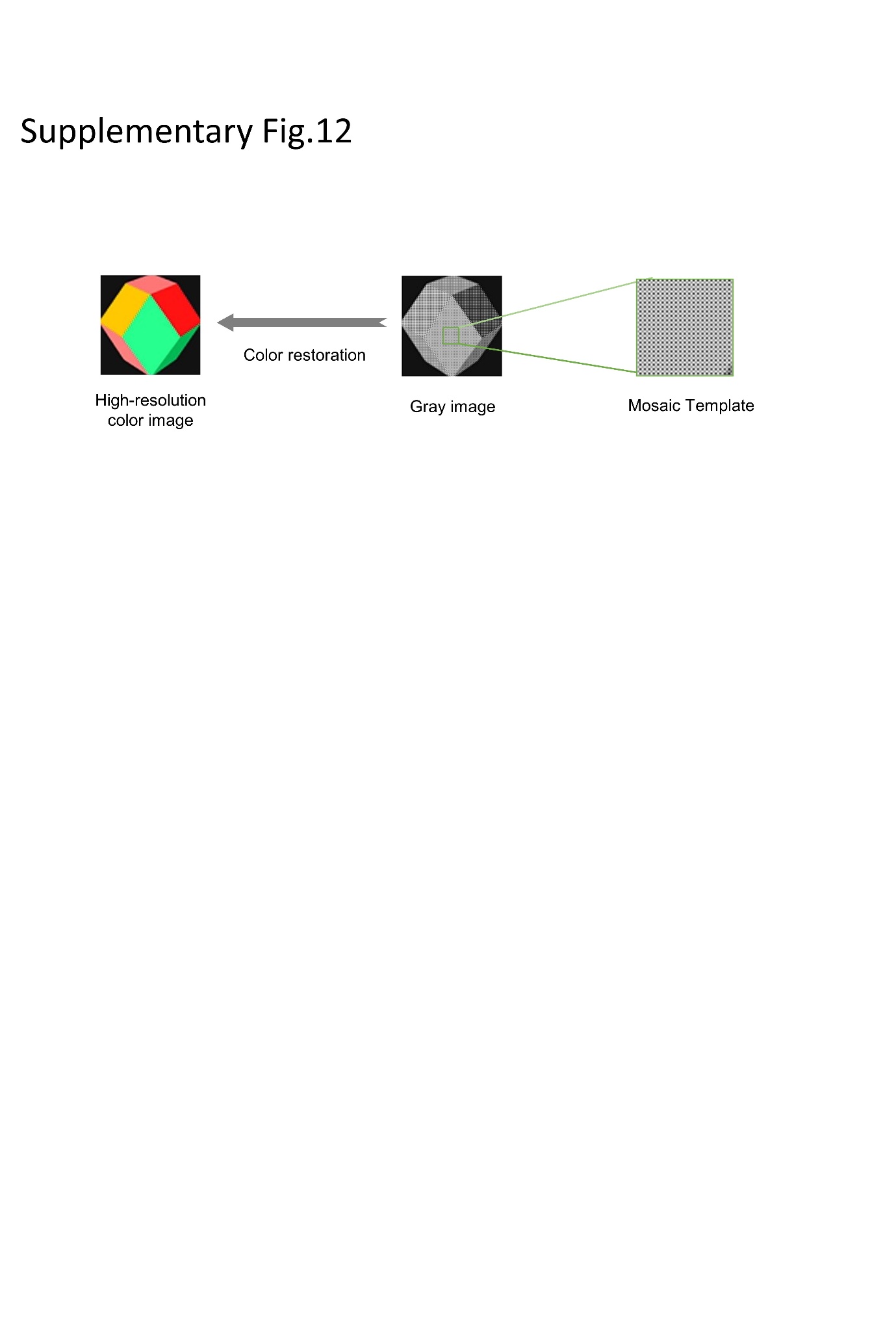


**Figure S12.**The schematic diagram of grayscale image color restoration principle.

Firstly, we measured the device's response coefficients to light intensity across different spectral channels. By projecting red, green, and blue patterns with the same resolution and amplitude onto a blank A4 paper, we gathered reflected light intensity values to determine the ratio of spectral responses. The obtained ratio of response coefficients was found to be μ_R_:μ_G_:μ_B_=0.77:1:0.69. This proportional relationship was then utilized during the iterative interpolation demosaicing process for color correction, to restore true color effects based on the detector's inherent color response. (Figure S13).


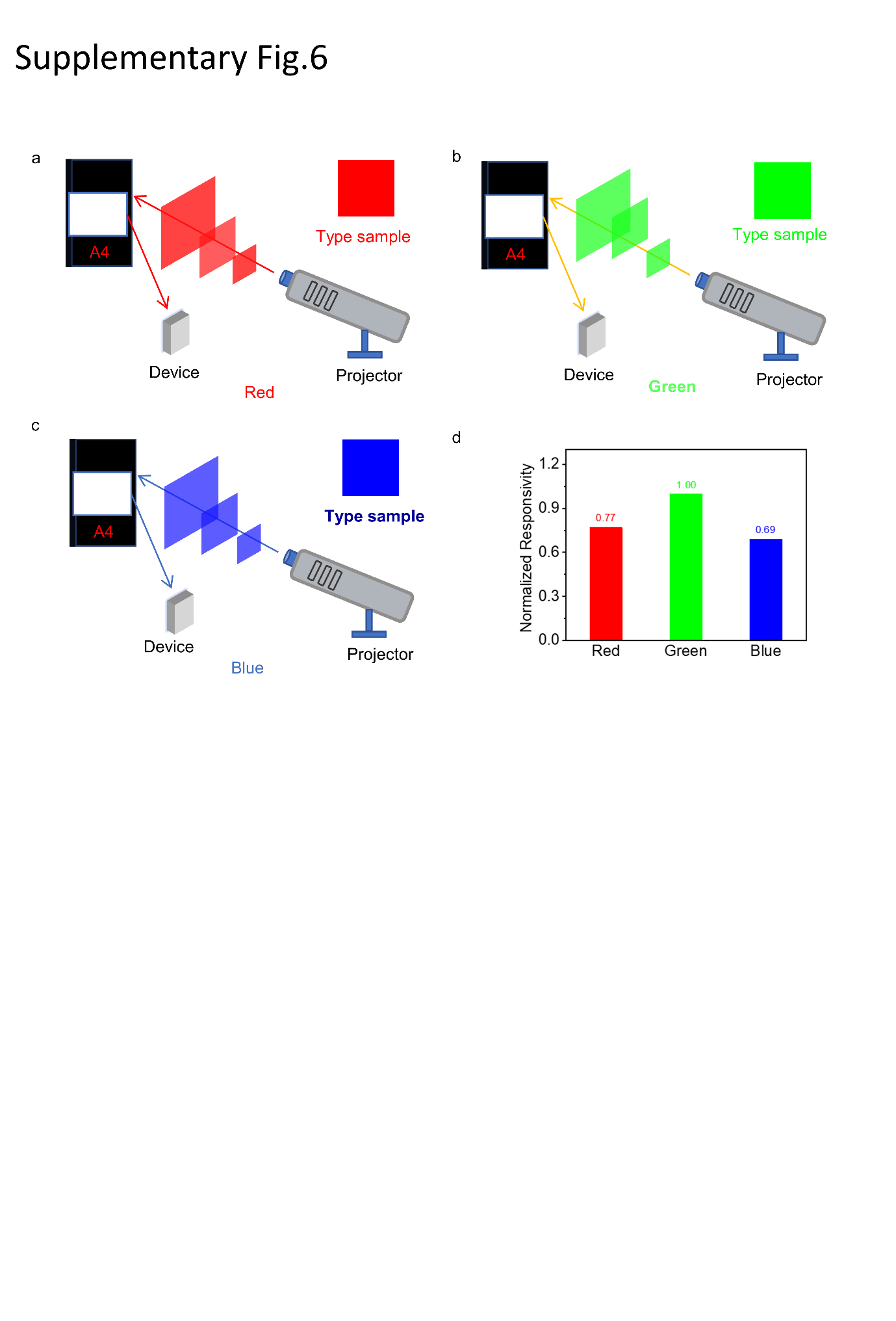


**Figure S13.** **Color correction based on the responsivity of the CsBi_3_I_10_-GTC photodetector. a-c,** Represent schematic diagrams of the device's R tests for red, green, and blue, **d,** Illustrates the device's responsivity to these three colors.

**Different Bayer color filter arrays in color imaging**

GRBG, RGGB, BGGR, and GBRG are four common arrangements of the Bayer color filter array (Figure S14a). These patterns, consisting of two green filters, one red filter, and one blue filter, align with the design principles of the classic Bayer filter. The color images generated by the Fourier color basis of these four patterns exhibit excellent imaging effects, aligning with human color perception principles. In Fourier single-pixel imaging, the uniformity of color distribution is crucial for accurate image acquisition and reconstruction. A uniform color distribution helps reduce spectral aliasing and interpolation errors. Based on simulated imaging results, GRBG has been selected as the template. On the other hand, GGRB, GRBW, GRBY, and RYYB are uncommon arrangements. Figure S14b illustrates the imaging effects of these four patterns. In the case of GGRB, the horizontal arrangement of GG can easily cause color overlap during the color interpolation and restoration process, leading to a decrease in image saturation. GRBW, with only three color channels, suffers from a loss of color information in the Fourier spectrum, resulting in poor image quality. While GRBY produces good color images, the introduction of a fourth color increases the amount of data required for color restoration, making imaging more challenging and time-consuming. Finally, RYYB lacks green, which is the most sensitive color perceived by the human eye. This leads to a yellowish tint in the generated images, distorting their original colors (Figure S14c).


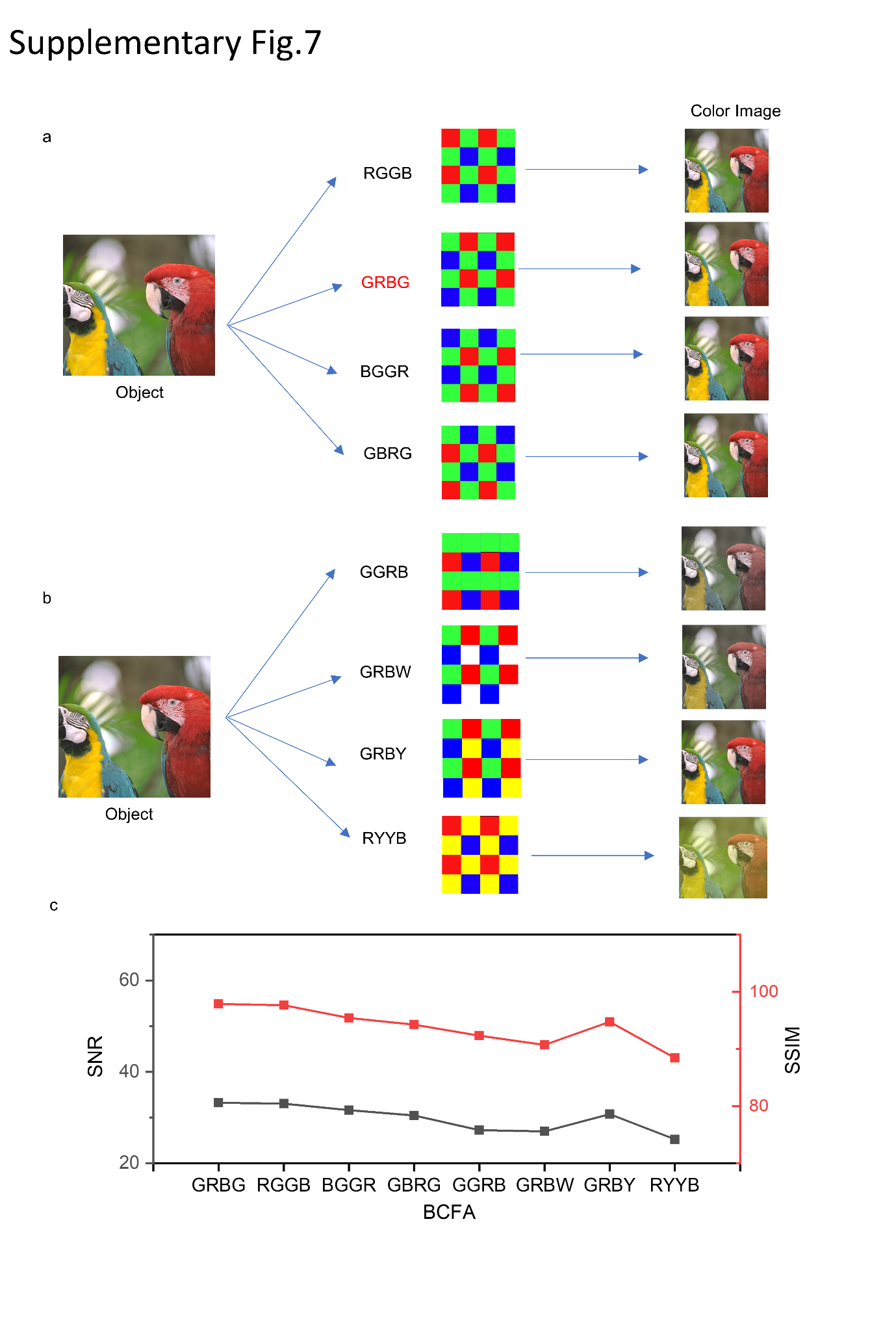


**Figure S14. Effect demonstration of** **different Bayer color filter arrays (BCFA) in color imaging. a,** Imaging effects of GRBG, RGGB, BGGR, and GBRG. **b,** Imaging effects of GGRB, GRBW, GRBY, and RYYB. **c,** Comparison of PSNR and SSIM of images obtained from different BCFA templates.

By decomposing the Bayer filter according to three different colored sub-filters, we can obtain red, green, and blue encoding templates (denoted as MR, MG, and MB respectively), which can be represented by the following matrix combination:


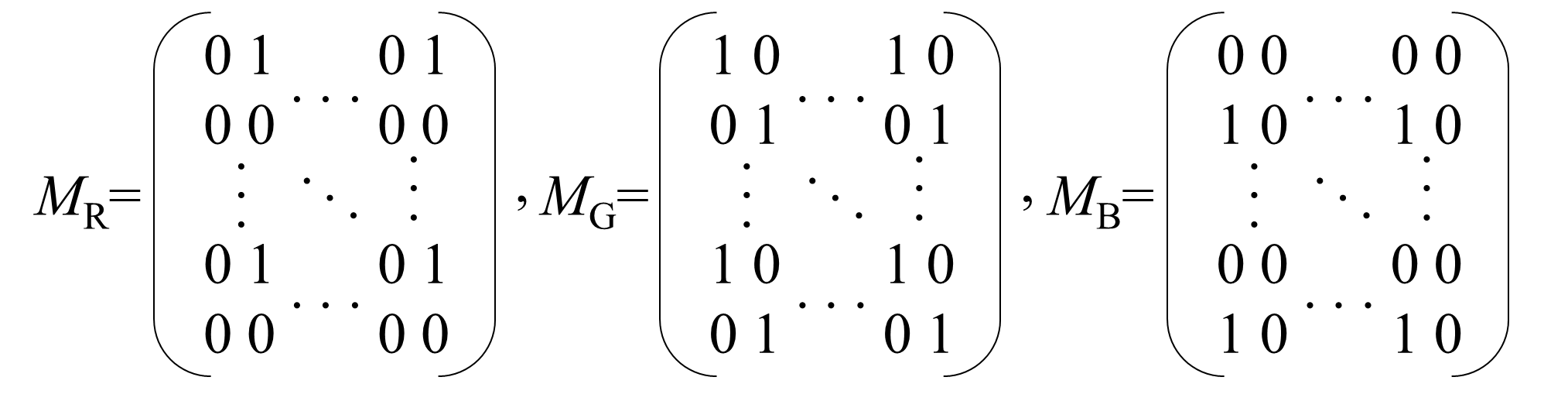


(21)

After performing two-dimensional Fourier transforms on the three-color encoding templates individually, it is observed that the encoding templates are actually composed of several pulses in the Fourier domain. These pulses are distributed along the edges and corners of the Fourier domain, which corresponds to the high-frequency region. This indicates that color is primarily concentrated in the high-frequency area of the spectrum. The Fourier transforms of the three templates can be represented by Eq. (22-24).

${\overset{\sim}{M}}_{R}=-\delta(0,-0.5)+\delta(-0.5,0)-\delta(-0.5,-0.5)$ (22)

${\overset{\sim}{M}}_{G}=\delta(0,-0.5)+\delta(-0.5,0)+\delta(-0.5,-0.5)$ (23)

${\overset{\sim}{M}}_{B}=\delta(0,-0.5)-\delta(-0.5,0)-\delta(-0.5,-0.5)$ (24)

When pulses from different color templates overlap in the Fourier domain, it means that these pulses share the same frequency components in the frequency domain. However, having the same frequency components does not imply that these signals are completely identical. Despite the possible overlap in frequency, there are differences in phase among the pulses from different color templates. Phase describes the position of a specific point (such as a peak or zero point) in the waveform cycle relative to a reference point. Due to phase differences, even with the same frequency components, these signals will manifest differently in the time domain. Although pulses from different color templates may have the same frequency components in the Fourier domain, they are linearly independent in that domain due to phase differences. Because of this linear independence, these signals can be treated as separate entities during processing and analysis. This implies that even with overlapping frequency components in the Fourier domain, it is possible to individually extract and process information for each color through appropriate signal processing techniques, without interference from other colors (Figure S15).

**
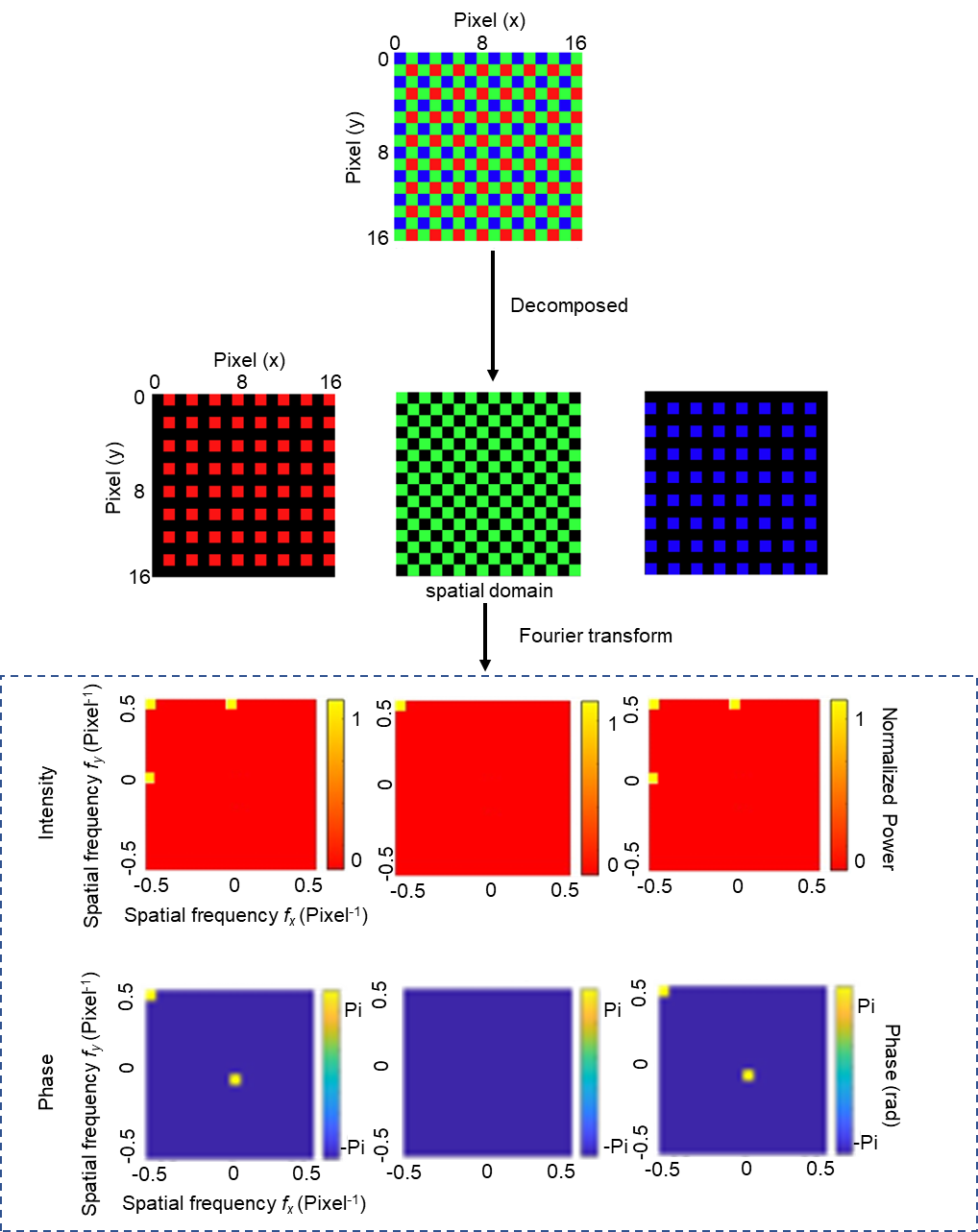
**

**Figure S15. Template design based on GRBG arrangement.** Split the GRBG template into three color templates and perform Fourier transformation.

The gray Fourier basis is modulated by a Bayer template to obtain color Fourier basis patterns. The low-frequency and high-frequency patterns of the color basis are shown in Figure S16. Since we chose a Bayer template mode with a GRBG arrangement for modulation, and because there are two green filters in each period, the Fourier basis appears green to the human eye. However, upon close inspection and local magnification, the periodic color arrangement can still be observed.


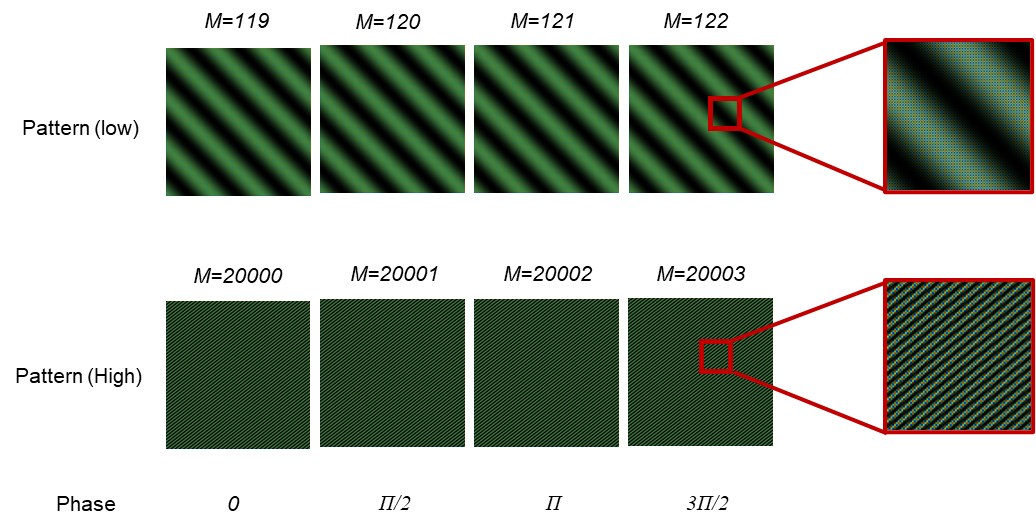


**Figure S16. Colored Fourier Spectrum.** Four Fourier basis colored patterns with the same spatial frequency (low and high frequencies).

**The influence of photodetector performance on imaging quality**

The curve in Figure S17. represents the current projection mode number (I-m) curve, clearly indicating that the obtained current intensity varies with the projection mode. Notably, within the four-step phase-shifting mode, specifically in the configurations of [*D_0_(fx, fy)-D_π_(fx, fy)] or [D_π/2(_f_x,_ f_y_) - D_3π/2_(f_x_, f_y_)*], the difference in current intensity demonstrates a remarkably high signal-to-noise ratio across both low and high frequencies, as illustrated in the corresponding figures. Consequently, the Fourier coefficients calculated based on the differences *[α_Re_ = D_0_(f_x_, f_y_) - D_π_(f_x_, f_y_)] and [α_im_ = j·[D_π/2_(f_x_, f_y_) - D_3π/2_(f_x_, f_y_)]]* are highly dependable.

**
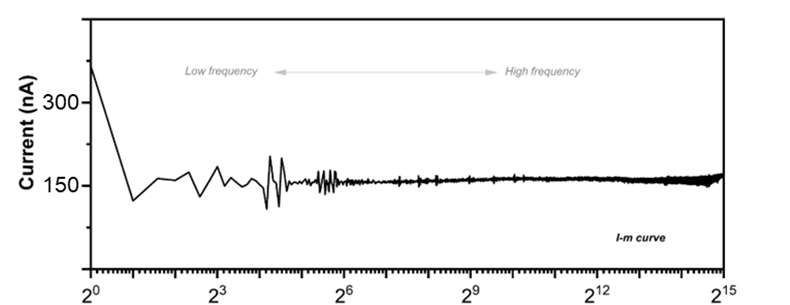
**

**Figure S17. I-m curve.** The I-m curve of CsBi_3_I_10_-GTC for imaging (128*128).

The current stability of the device is crucial to the possibility and quality of imaging. Figure S18 shows the I-t curves of CsBi_3_I_10_-GTC and CsBi_3_I_10_ photodetectors measured under different intensities ranging from 0 to 90 µW cm^-2^. The current fluctuation of CsBi_3_I_10_ is significantly higher than that of the CsBi_3_I_10_-GTC film with additives. This indicates that introducing additives improves the device's current stability, which plays an important role in improving the imaging quality of the device and its imaging performance under low-light conditions.

The photocurrent has only slight differences, especially when m increases. Therefore, when the mode is switched, the fluctuation of the photocurrent should be small enough to perceive the slight changes in diffuse reflection, which also means that the photocurrent mode (I-m) curve should have obvious steps. Otherwise, the real part of the Fourier inverse coefficient will not be accurate, which may bring noise and even image distortion. Therefore, the standard deviation (SD) of the photocurrent should meet the following conditions as Eq. (25):

$SD\ll D_{\Phi1}(f_{x},f_{y})-D_{\Phi2}(f_{x},f_{y})$ (25)

Now let's assume that when there is only a small difference in light intensity between different modes, SD and R are the same, that is, R = R_1_ = R_2_. Now it can be rewritten as Eq. (26).

$\begin{matrix} & SD<R_{1}kE_{\Phi1}(f_{x},f_{y})-R_{2}kE_{\Phi2}(f_{x},f_{y}) \\ & =R[kE_{\Phi1}(f_{x},f_{y})-kE_{\Phi2}(f_{x},f_{y})] \end{matrix}$ （26）

Divide both sides by R to get Eq. (27).

$SD/R\ll kE_{\Phi1}(f_{x},f_{y})-kE_{\Phi2}(f_{x},f_{y})$ （27）

The left side of the equation represents the inherent characteristics of the photodetector, while the right side reflects changes in light intensity, independent of the photodetector itself. Based on this, we can introduce a new metric, the photodetector quality factor, denoted as Q=SD/R, for our imaging technology. Here, R for the responsivity, and SD for the standard deviation of the photocurrent under specific light intensities. The Q parameter, which shares the same dimensionality as light intensity, signifies the minimum detectable change in light intensity by the photodetector. Since the photocurrent is a superposition of both photocurrent and dark current, the intensities of these two components critically influence the usability of single photodetector imaging. Consequently, it can be anticipated that measures aimed at reducing the fluctuations in dark current and photocurrent, or enhancing the responsivity rate of the photodetector, will lead to improved image quality.


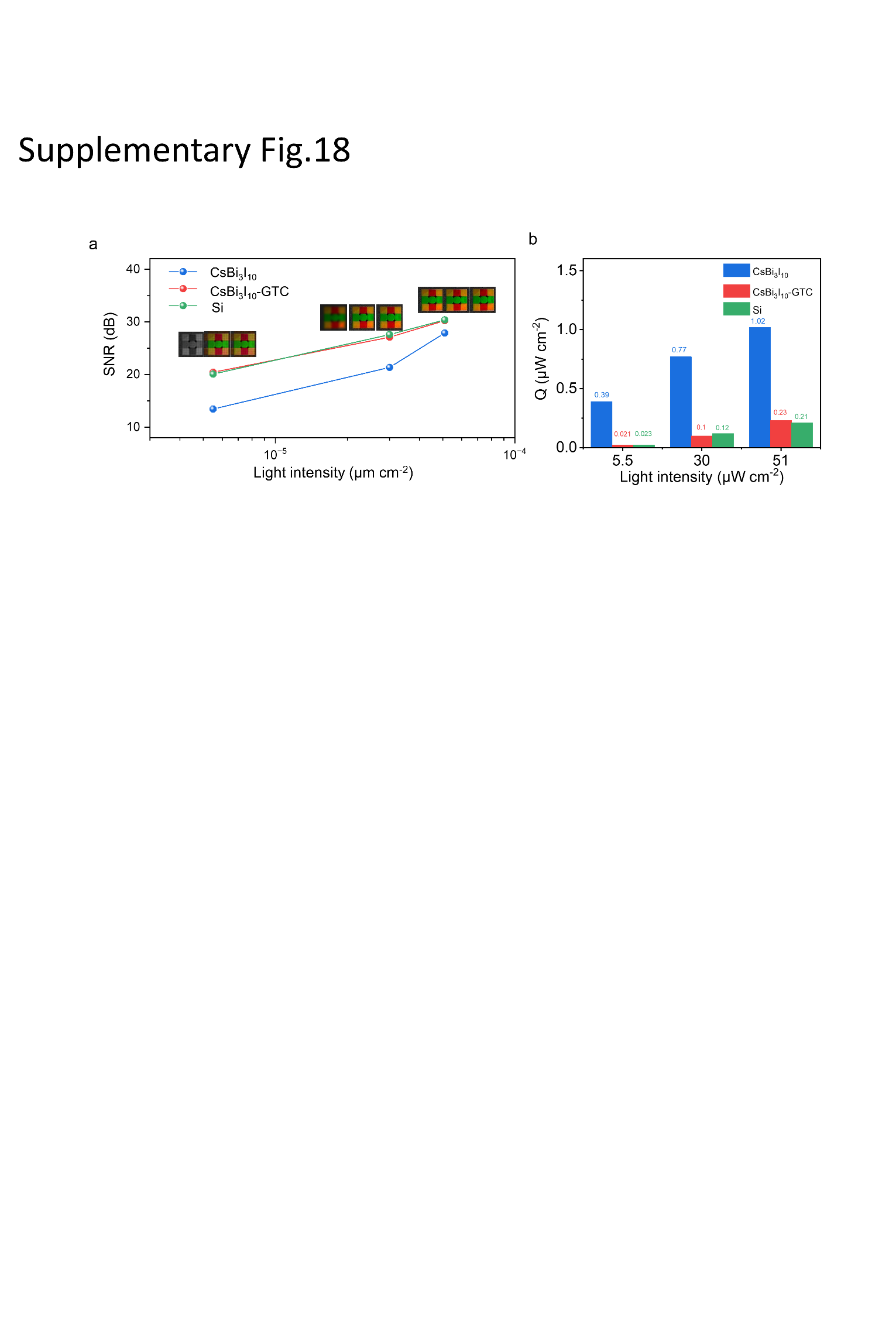


**Figure S18.** Comparison of imaging quality among three devices (CsBi_3_I_10_, CsBi_3_I_10_-GTC, and SiPMs). a. Comparison of imaging quality among the three devices under different light intensities (Left: CsBi_3_I_10_, Middle: CsBi_3_I_10_-GTC, and Right: SiPMs). b. Comparison of calculated Q values among the three devices under different light intensities.

We compared the imaging effects of CsBi_3_I_10_, CsBi_3_I_10_-GTC, and Si photodetector under different light intensities. By comparing the SNR of the imaging produced by the three devices under different light intensities, as shown in Figure S19a, we found that under the same conditions, the imaging effect of the CsBi_3_I_10_-GTC photodetector is better than that of the CsBi_3_I_10_ photodetector, indicating that the CsBi_3_I_10_-GTC photodetector has a good imaging effect. Meanwhile, under different light intensities, the imaging effect of the CsBi_3_I_10_-GTC photodetector is comparable to that of commercially available SiPMs. Especially under low light conditions, it even has a better imaging effect than SiPMs. Additionally, we calculated the Q, where a smaller Q indicates greater suitability for imaging under the same conditions. The Q values for the three devices under varying light intensities align with the comparison results of the SNR values, further highlighting the excellent imaging performance of the CsBi_3_I_10_-GTC photodetector Figure S19b.


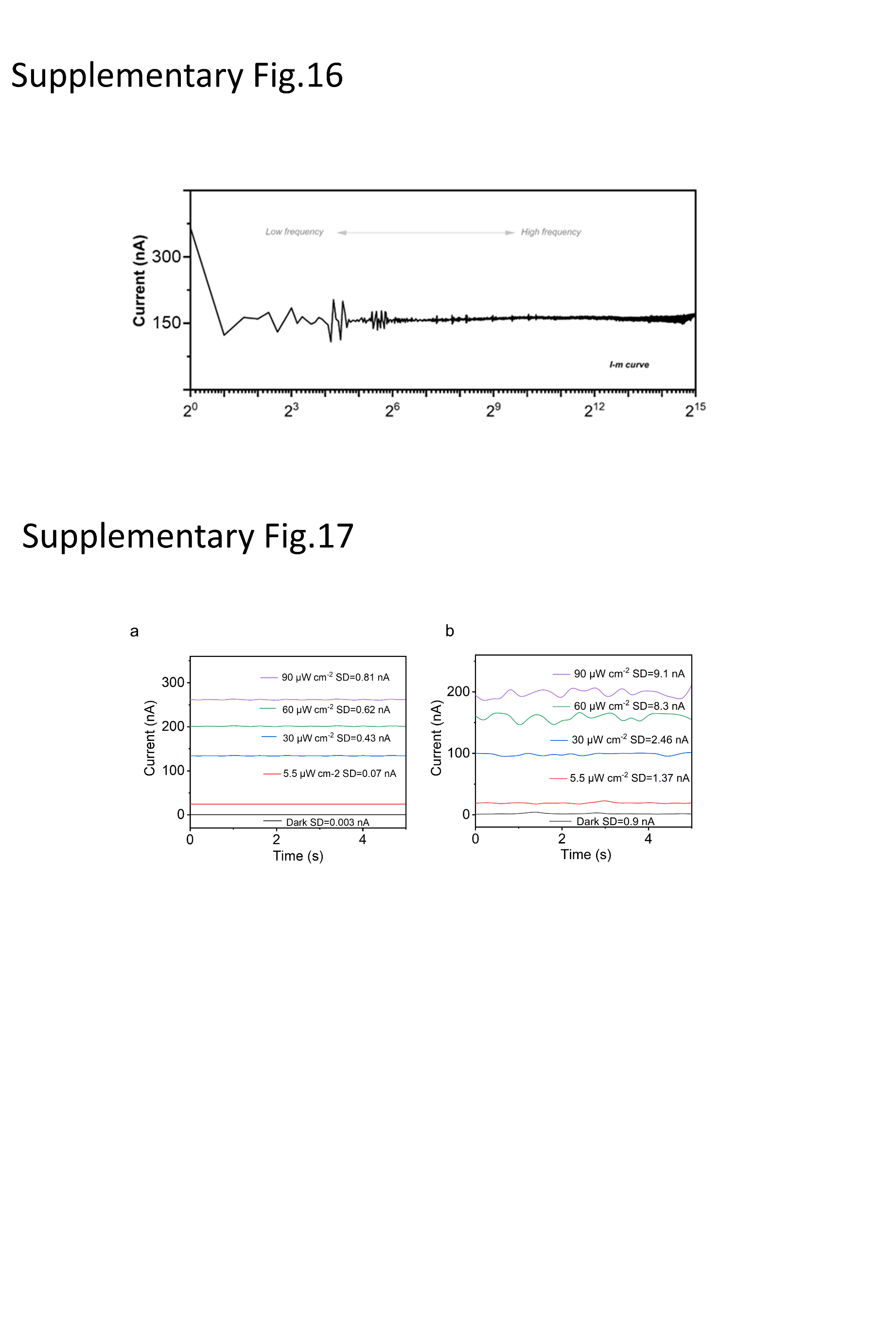


## Figure S19. Current fluctuation. a, The I-t curves of the CsBi_3_I_10_-GTC photodetector were obtained under the illumination of white lights with varying intensities ranging from 0 to 90 µW cm^-2^. b, The I-t curves of the CsBi_3_I_10_ photodetector were obtained under the illumination of white lights with varying intensities ranging from 0 to 90 µW cm^-2^.

Figure S20 is a cross-sectional view of the hemispherical device. Through SEM characterization of the perovskite layer on the surface of the hemispherical device, it is found that the perovskite layer prepared by spraying has good uniformity^[9]^.


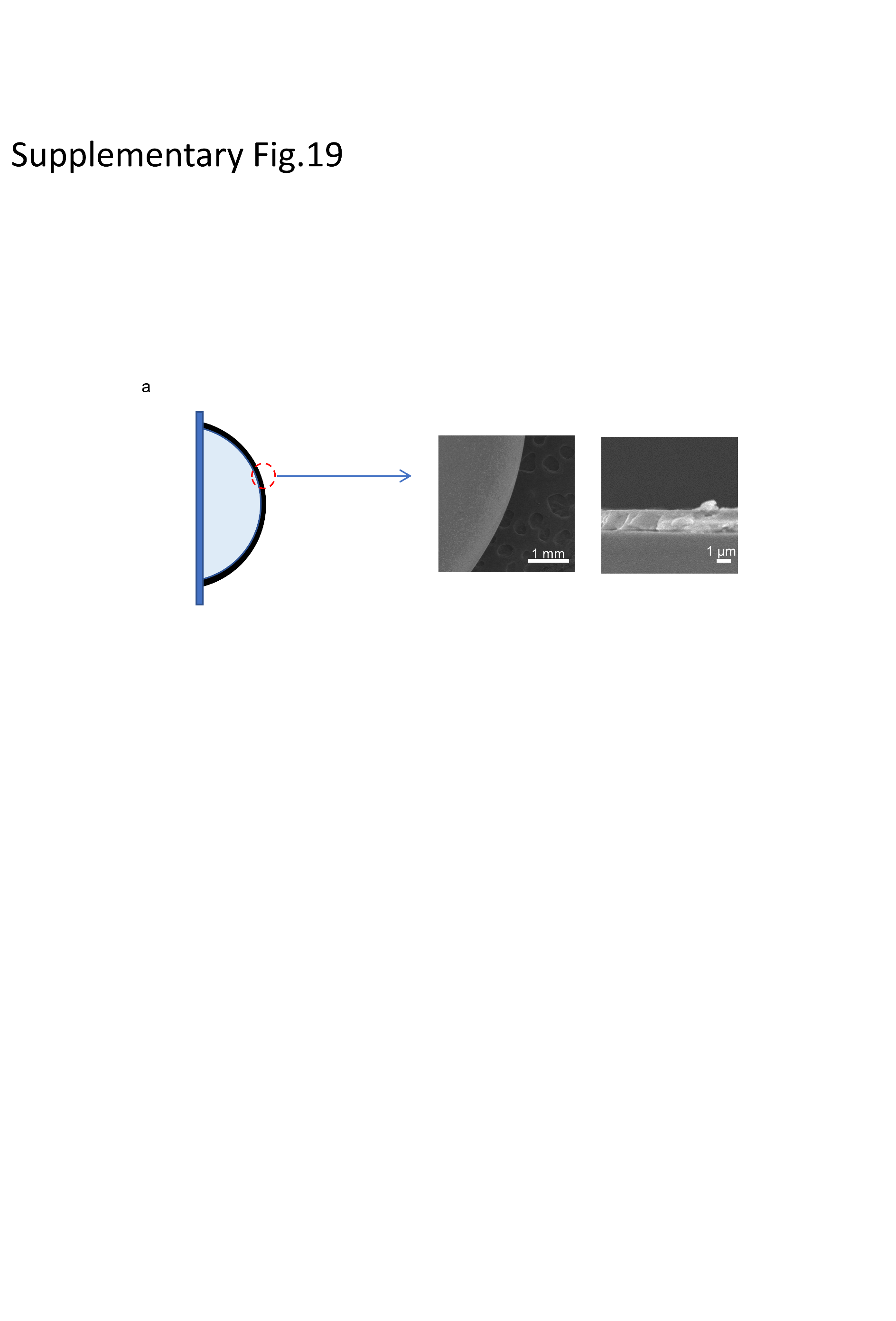


**Figure S20. SEM image of the hemispherical device.** **a,** Local image of the perovskite thin film on the surface of the perovskite hemispherical device.

There is a significant difference between hemispherical detectors and planar detectors in receiving illumination. As shown in Figure S21, I_⊥_ represent the vertical component of the incident light I_O_, while Psi (ψ) and Phi (φ) are used to measure the incident angle of light relative to a planar surface and a hemispherical surface, respectively. For hemispherical objects, as the incident angle changes, I_⊥_ does not vary with the angle. However, the distribution of I_⊥_ on the surface of the sphere is different, and it can be expressed using the following Eq. (28).

$I_{\perp}=\cos\psi I_{O}$ (28)

Here, ψ is the angle formed by the light ray passing through the center of the sphere and the perpendicular line to the base plane of the hemispherical surface. For planar objects, the irradiance on the plane is uniform, so I_⊥_ depends on the angle between the plane and the incident light. Therefore, I_⊥_ can be calculated using the following Eq. (29).

$I_{\perp}=\cos\varphi I_{O}$ (29)

Here, φ is the angle between the plane and the incident light. The effective luminous flux on the hemispherical surface can be calculated using Eq. 30.

$\boldsymbol{\Phi}=\mathbf{I}_{\mathbf{0}}\cdot\mathbf{S}=\iint\cos\psi I_{O}dS$ (30)

where dS represents the differential area on the hemispherical surface, which can be calculated using Eq. 31.

$dS=r^{2}\sin\theta d\theta d\phi$ (31)

Here, r, θ, φ are the coordinate parameters in spherical coordinates, so the luminous flux on the spherical surface can ultimately be obtained using the following formula.

$\Phi=\iint I_{O}r^{2}\sin\psi\cos\theta d\theta d\phi$ (32)

Ideally, a hemispherical photodetector can respond to incident light from almost 360°^[10]^. As can be seen from Figure S21, under the same projected area, the equivalent luminous flux of a hemispherical photodetector is always greater than that of a planar detector.


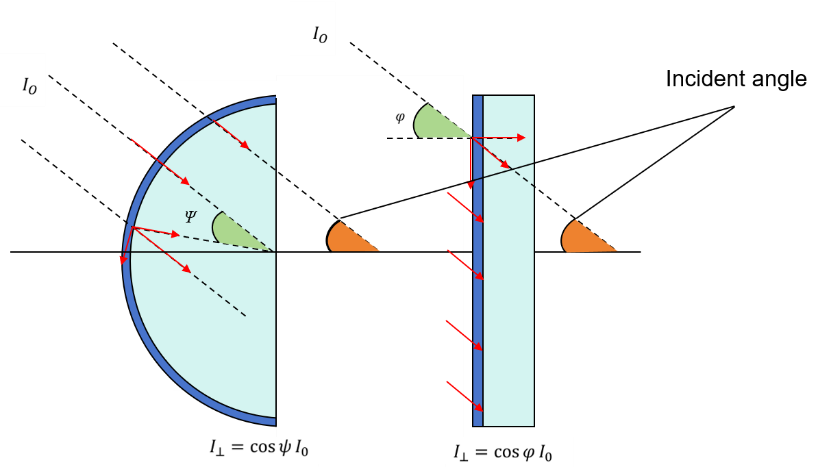


**Figure S21.** Schematic diagram of the vertical and parallel components of incident light on a hemispherical surface and a planar surface. The incident light (red arrow) is decomposed into components parallel and perpendicular to the surface. The angles Ψ and φ are used to calculate the angle of the incident light.


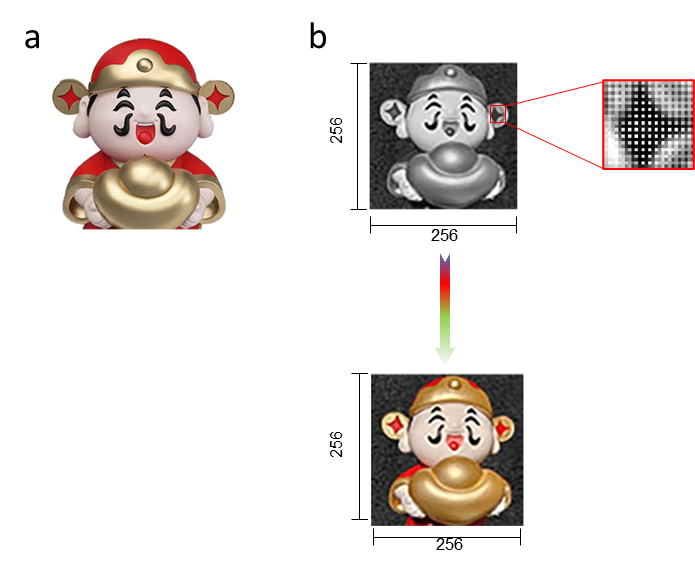


**Figure S22. High-resolution color imaging. a,** Real photos of toys. **b,** The process of Fourier single-pixel color imaging.

By comparing the imaging quality of hemispherical photodetectors and planar photodetectors at different angles under different light intensities (30 μW cm^-2^ and 50 μW cm^-2^), it is found that the imaging quality of hemispherical photodetectors is always better than that of planar photodetectors under the same conditions (Figure S23). This is because the design of hemispherical photodetectors allows them to capture and focus light more effectively, which increases their equivalent luminous flux. As a result, the equivalent luminous flux of hemispherical photodetectors is always greater than that of planar photodetectors.


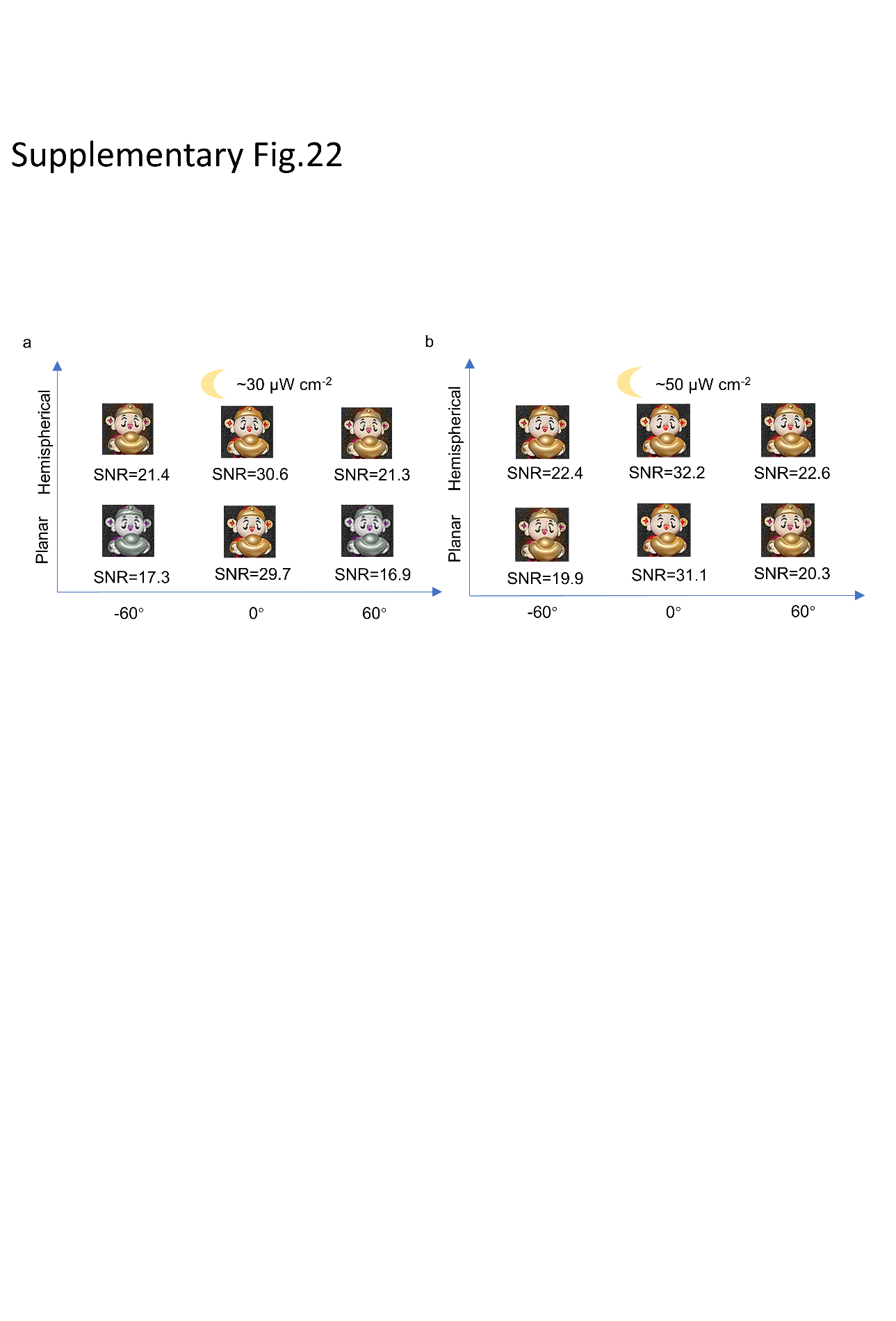


**Figure S23. Imaging effect demonstration under different conditions.** Comparison of imaging quality between hemispherical and planar photodetectors at different angles under different light intensities (**a,** 30 μW cm^-2^ and **b,** 50 μW cm^-2^).

The current stability of SiPMs and CsBi_3_I_10_-GTC photodetectors at different temperatures is compared by calculating the standard deviation of the current. When the temperature reaches 85 °C, SiPMs reach their operating temperature, and their current fluctuations significantly increase compared to those at normal temperature. However, the current fluctuations of the CsBi_3_I_10_-GTC device are relatively minor. When the temperature rises to 100 °C, the current fluctuations of SiPMs become very intense. According to the calculated Q value, it is found that the Q value increases significantly, indicating that the device can no longer meet the requirements for imaging. However, the change in the Q value of CsBi_3_I_10_-GTC at 100 °C is not significant. This also indirectly indicates that the CsBi_3_I_10_-GTC device we prepared is suitable for imaging at high temperatures (Figure S24).

**
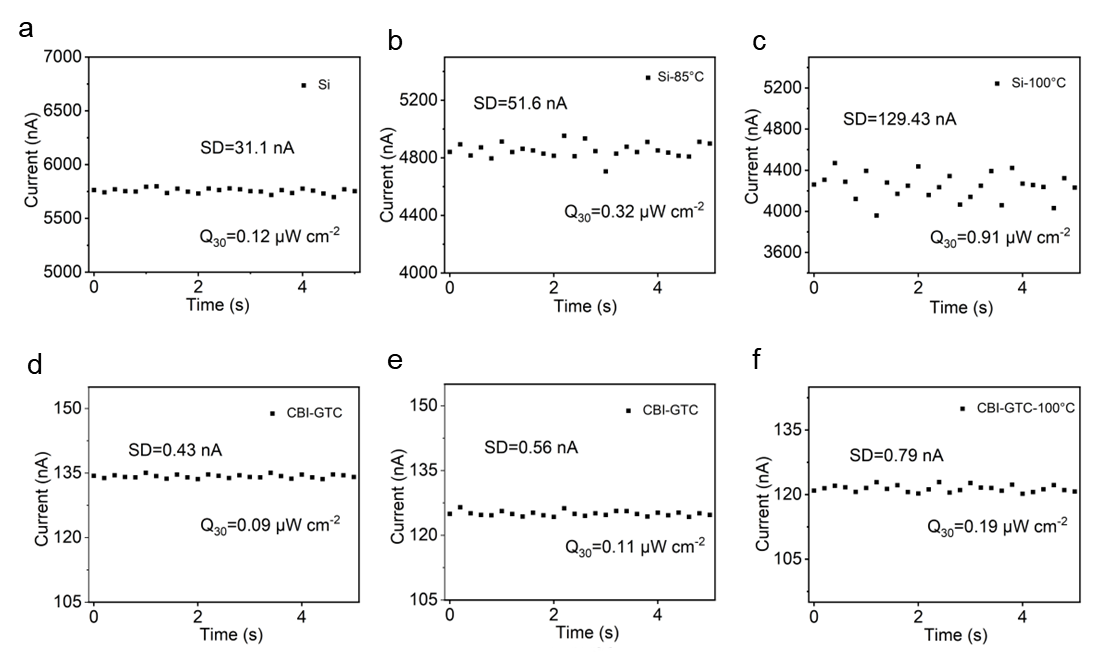
**

**Figure S24. Comparison of current stability under different conditions. a-c,** Current stability diagrams of Si material over time at different temperatures (0 °C, 85 °C and 100°C); **d-e,** Current stability diagrams of CsBi_3_I_10_-GTC material over time at different temperatures (0 °C, 85 °C and 100 °C).


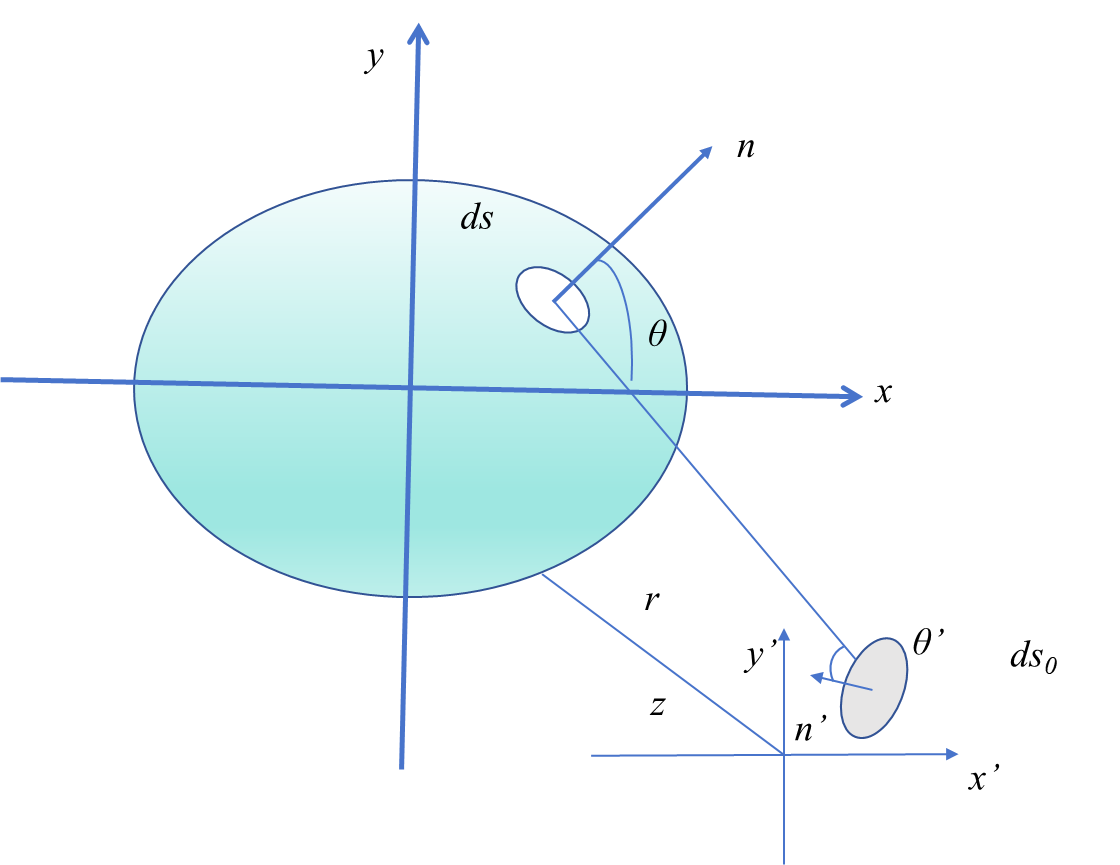


**Figure S25.** Schematic diagram of the luminous flux received by a photodetector from diffuse reflection.

#

# Supplementary References

[1] H. M. Ren, G. Deng, P. Zhou, X. Kang, Y. Zhang, J. Ni, Y. Zhang, Y. Wang, *Journal of Biomedical Optics* **2022**, 27.

[2] R. He, Z. Weng, Y. Zhang, C. Qin, J. Zhang, Q. Chen, W. Zhang, *Optics Express* **2021**, 29.

[3] J. Huang, D. Shi, K. Yuan, S. Hu, Y. Wang, *Optics Express* **2018**, 26.

[4] Z. Zhang, X. Ma, J. Zhong, *Nature Communications* **2015**, 6.

[5] G. Wang, H. Deng, M. Ma, X. Zhong, *Optics Letters* **2023**, 48.

[6] J. Wang, Y. Pan, Z. Zhou, Q. Zhou, S. Liu, J. Zhang, C. Shi, R. Chen, Z. Zhao, Z. Cai, X. Qin, Z. Zhao, Z. Yang, Z. Liu, W. Chen, *Advanced Energy Materials* **2024**.

[7] Y. Liu, Z. Ji, *Applied Physics Letters* **2023**, 122.

[8] F. Zhang, Y. Gao, D. Wang, P. Lu, X. Wang, M. Lu, Y. Wu, P. Chen, J. Hu, X. Bai, Z. Wu, D. Zhou, D. Liu, L. Xu, B. Dong, H. Song, Y. Zhang, *Nano Energy* **2024**, 120.

[9] X. Feng, Y. He, W. Qu, J. Song, W. Pan, M. Tan, B. Yang, H. Wei, *Nature Communications* **2022**, 13.

[10] H. Jin, R. Zhang, S. Liu, Z. Zheng, Y. Zheng, *IEEE Transactions on Biomedical Engineering* **2019**, 66, 1810.

1. *Email: hweichem@jlu.edu.cn [↑](#footnote-ref-1)
